# *Rosenbergiella meliponini* D21B Isolated from Pollen Pots of the Australian Stingless Bee *Tetragonula carbonaria*

**DOI:** 10.3390/microorganisms11041005

**Published:** 2023-04-12

**Authors:** Anthony J. Farlow, Darshani B. Rupasinghe, Khalid M. Naji, Robert J. Capon, Dieter Spiteller

**Affiliations:** 1 Chemical Ecology/Biological Chemistry, Department of Biology, University of Konstanz, Universitätsstraße 10, 78457 Konstanz, Germany; 2 Centre for Drug Discovery, Institute for Molecular Bioscience, The University of Queensland, 306 Carmody Road, Brisbane, QLD 4072, Australia

**Keywords:** genome, mass spectrometry, microbial symbionts, 2-phenylethanol, phylogeny, secondary metabolite, vitamins, volatile compounds

## Abstract

*Rosenbergiella* bacteria have been previously isolated predominantly from floral nectar and identified in metagenomic screenings as associated with bees. Here, we isolated three *Rosenbergiella* strains from the robust Australian stingless bee *Tetragonula carbonaria* sharing over 99.4% sequence similarity with *Rosenbergiella* strains isolated from floral nectar. The three *Rosenbergiella* strains (D21B, D08K, D15G) from *T. carbonaria* exhibited near-identical 16*S* rDNA. The genome of strain D21B was sequenced; its draft genome contains 3,294,717 bp, with a GC content of 47.38%. Genome annotation revealed 3236 protein-coding genes. The genome of D21B differs sufficiently from the closest related strain, *Rosenbergiella epipactidis* 2.1A, to constitute a new species. In contrast to *R. epipactidis* 2.1A, strain D21B produces the volatile 2-phenylethanol. The D21B genome contains a polyketide/non-ribosomal peptide gene cluster not present in any other *Rosenbergiella* draft genomes. Moreover, the *Rosenbergiella* strains isolated from *T. carbonaria* grew in a minimal medium without thiamine, but *R. epipactidis* 2.1A was thiamine-dependent. Strain D21B was named *R. meliponini* D21B, reflecting its origin from stingless bees. *Rosenbergiella* strains may contribute to the fitness of *T. carbonaria.*

## 1. Introduction

Bees are crucial as pollinators in ecosystems and agriculture. As with other insects, microorganisms are associated with bees that deliver important services to their host [1,2]. For example, microbial amylase production is required for the processing of plant nectar [3], microbial proteases support protein digestion [4], short-chain fatty acids are provided by bacteria [2], and microorganisms stimulate hormone production [5,6]. It has also been proposed that bees benefit from vitamins produced by microbial symbionts [7]. The bacterium *Snodgrassella*, which lives in the hindguts of bumblebees and honeybees, produces aromatic amino acids by the Shikimate pathway [8]. Although this does not appear to be of direct nutritional benefit to bees because amino acids are believed to be absorbed by the midgut [5], it supports the growth of other organisms in the hindgut that lack the Shikimate pathway [9]. Certain Bacilli appear to contribute to the protection of their hosts against pathogens [10,11,12,13], as do various strains of *Lactobacillus* and *Bifidobacterium* [14]. Beneficial bee symbionts are not restricted to the digestive system. Bee pollen (sometimes known as bee bread) is a microbially fermented product obtained from the food comb or honey pots of many bees, both social and solitary [15,16,17,18,19]. In order to investigate the role of microbial symbionts of the Australian stingless bee *Tetragonula carbonaria* (formerly *Trigona carbonaria*), we isolated and characterized microorganisms from a *T. carbonaria* hive located in Brisbane, Australia.

While little data exist on Australian stingless bee population trends, *T. carbonaria* appears to be relatively free of pests and pathogens and is an easy bee to maintain [20]. So far, only one microbial pathogen has been described [21]. This is in sharp contrast to the multiple stressors of honeybees, including parasites that act as vectors for viruses and bacterial infections [22,23,24,25,26]. These stressors of honeybees, along with the decline in pollinator populations more generally, could potentially threaten food security [27]. Thus, *T. carbonaria* attracted our interest, and we initiated studies to reveal why it has been largely unaffected by the pollinator decline. One possibility for this robustness may be that microbial symbionts protect *T. carbonaria* against pathogens.

Here, we describe the isolation of three *Rosenbergiella* bacteria from the Australian stingless bee *T. carbonaria.* The *Rosenbergiella* isolates were characterized by phylogenetic analysis, and the genome of one isolate was sequenced. Moreover, the physiology, biochemistry, and characteristic metabolites of the *Rosenbergiella* isolates were studied.

## 2. Materials and Methods

### 2.1. Chemicals and Media Components

Unless otherwise specified, all chemicals and media components were from Carl Roth GmbH, Karlsruhe, Germany.

### 2.2. Microorganisms

*Rosenbergiella epipactidis* 2.1A (strain number LMG 27956) was obtained from the Belgian Coordinated Collection of Microorganisms (BCCM). *Rosenbergiella* sp. D08K, *Rosenbergiella* sp. D15G, and *Rosenbergiella meliponini* D21B were isolated from a domesticated hive of the stingless bee *Tetragonula carbonaria* located in Brisbane, Australia (27°33′ S, 152°56′ E), in 2017.

### 2.3. Deposition of Rosenbergiella meliponini D21B

*Rosenbergiella meliponini* D21B was deposited at the Belgian Coordinated Collection of Microorganisms, Belgium (strain number: LMG 32782), and The National Collection of Industrial, Food and Marine Bacteria, United Kingdom (strain number: NCIMB 15457).

### 2.4. Isolation of Microorganisms from T. carbonaria

Media used to cultivate *Rosenbergiella* are listed in the Supplementary Materials.

In order to isolate microorganisms from the *T. carbonaria* hive, yellow granular pollen was streaked onto pollen agar and incubated at 28 °C. After 3 days, the resulting colonies were re-streaked on J agar [28]. Four rounds of re-streaking were performed on J agar to isolate pure microorganisms.

*Rosenbergiella* sp. D08K and *Rosenbergiella* sp. D15G were isolated from the hindgut of a dissected stingless bee. A whole *T. carbonaria* worker bee was carefully opened using sterilized tweezers, and the digestive tract was divided into three segments. The digestive tract occurs as three distinguishable segments- a thin tube and a larger crop, the midgut, and the hindgut. These segments were separated using a second pair of sterile tweezers that did not touch the bee exterior. Each segment was added into 100 μL of J medium and crushed with a sterile pipette tip (200 μL) to make a homogeneous mixture. The J medium mixture was incubated at 37 °C for 30 min at 225 rpm. The mixture (1.0 mL) was spread over a J agar plate and incubated at 28 °C for 2 days, yielding about 100 colonies. Individual colonies were picked with sterile toothpicks and transferred to fresh J agar plates, and sub cultured four times on J agar to obtain pure colonies.

For glycerol stocks, pure isolates were grown overnight in liquid J broth at 28 °C. A 400 μL aliquot of culture broth was mixed with sterile glycerol solution (400 μL, 80%), snap-frozen in liquid nitrogen, and stored at −78 °C.

### 2.5. Cultivation of Microorganisms from T. carbonaria

In order to determine which vitamins are required for growth, *Rosenbergiella* were grown in a minimal medium [29]. This medium was supplemented with seven B-group vitamins and precursors adapted from Pfennig [30] (Supplementary Materials). Because genes may encode for pyridoxal, and biotin and folic acid biosynthesis were identified in the *R. meliponini* D21B draft genome, the vitamin mixture was limited to thiamine, nicotinic acid, pantothenic acid, and cobalamin. These vitamins were selectively removed one at a time from the growth medium until the essential vitamins were established for each organism.

Each vitamin assay was carried out as follows: A 2 mL aliquot of minimal medium supplemented with vitamin solution was transferred to a culture tube and inoculated with *Rosenbergiella* grown on no-salt lysogeny broth (NSLB) agar [31] supplemented with 10% sucrose. The mixture was incubated for 2 days (28 °C, 150 rpm). A 100 μL aliquot of this culture was added to the same medium (20 mL) in a sterile Erlenmeyer flask and incubated at 28 °C (150 rpm). The optical density at 600 nm was recorded after 2 days.

### 2.6. Genome Sequencing and Genome Assembly

A single colony of *R. meliponini* D21B was used to inoculate 3 × 5 mL of J medium, which was incubated at 28 °C (150 rpm) overnight. The frozen pellet of *R. meliponini* D21B was submitted for genomic DNA isolation and genomic sequencing by Eurofins Genomics (Konstanz, Germany). The draft genome was sequenced and assembled by Eurofins Genomics using Illumina HiSeq (2 × 150 bp paired-end, inview genome resequencing).

### 2.7. Annotation of the R. meliponini D21B Genome

The draft genome was annotated with the Integrated Microbial Genomes (IMG) annotation pipeline v.5.0.20 using the following programs and databases: GeneMark.hmm-2 v1.05; INFERNAL 1.1.3 (November 2019); Prodigal v2.6.3, tRNAscan-SE v.2.0.6 (May 2020); the annotation algorithm: lastal 1066, HMMER 3.1b2, signalp 4.1, decodeanhmm 1.1g. The genome annotation by IMG was aided by the support database(s), including Rfam 13.0, IMG-NR 20190607, SMART 01 06_2016, COG 2003, TIGRFAM v15.0, SuperFamily v1.75, Pfam v30, Cath-Funfam v4.2.0 [32].

### 2.8. Genomic DNA Isolation

Genomic DNA was prepared using the protocol described by Wright [33]. Briefly, a bacterial culture (3 mL) was grown overnight in J broth (28 °C, 150 rpm). The cells were pelleted by centrifugation (8000 rpm, 2 min) and resuspended in 500 μL NaCl-Tris-EDTA buffer (75 mM NaCl; 25 mM EDTA; 20 mM Tris). Lysozyme (50 mg/mL, 20 μL) was added, and the mixture was incubated at 37 °C for 10 min. Proteinase K (20 mg/mL, 20 μL) was added, and the resuspended pellet was incubated at 55 °C for a further 60 min. Sodium chloride (5 M, 200 μL) was added, and the solution was washed with chloroform:isoamyl alcohol (24:1, 2 × 400 μL). DNA was precipitated with isopropyl alcohol at 0 °C. The DNA was pelleted by centrifugation (13,000 rpm, 15 min, 2 °C). The pellet was washed with 70% ethanol in water (0 °C, 200 μL), and the DNA was re-pelleted (13,000 rpm, 2 min, 2 °C). The DNA pellet was re-dissolved in 50 μL sterile deionized water and stored at −20 °C.

### 2.9. 16S rDNA Amplification by PCR

16*S* rRNA gene regions were amplified using the primers 8F (AGAGTTTGATCCTGGCTCAG) [34] and 1492r (GGTTACCTTGTTACGACTT) [35] (Eurofins Genomics, Ebersberg, Germany) in 50 μL reaction volumes containing 200 μM each dNTP (10 mM, 1 μL each), forward and reverse primers (10 μM), 1 uL of S7 Fusion High-Fidelity DNA polymerase (Biozym, Oldendorf, Germany), 10 μL of 5 × GC buffer, magnesium chloride (50 mM, 5 μL), dimethyl sulfoxide (4 μL), and 300 ng of genomic DNA template.

PCR conditions: Samples were denatured at 98 °C for 30 s and followed by 30 cycles of 10 s at 98 °C (denaturation), 30 s at 57 °C (annealing), and 45 s at 72 °C (extension), with a final extension at 72 °C for 10 min. PCR products were purified using 1.5% agarose gel electrophoresis. Bands of ~1500 bp were cut from the gel, and the DNA was extracted using the peqGOLD gel extraction kit (VWR, Darmstadt, Germany). Purified DNA was ligated into the linearized pJET1.2 vector using the CloneJET PCR cloning kit (ThermoFisher Scientific, Darmstadt, Germany). *E. coli* Top10 cells were transformed with the resulting plasmid, and colonies viable on LB Luria agar with 50 μg/mL ampicillin were grown in LB Luria medium with 50 μg/mL ampicillin. The overexpressed plasmid was extracted with HiYield plasmid mini prep extraction kit (Süd-Laborbedarf, Gauting, Germany) and sequenced by Eurofins Genomics (Cologne, Germany) using the pJET 1.2 primers.

### 2.10. Phylogenetic Analysis

The 16*S* rRNA genes of *Rosenbergiella* isolates D21B, D08K, and D15G were sequenced as described above. The housekeeping genes rpoB, atpD, and gryB were extracted from the draft genome of *R. meliponini* D21B. Homologs of all the above mentioned genes from other *Rosenbergiella* strains were downloaded from the National Center for Biotechnology Information (NCBI) nucleotide database (https://www.ncbi.nlm.nih.gov/nucleotide/; accessed on 10 November 2020).

Gene sequences were aligned using Muscle, and phylogenetic trees were constructed based on the neighbor-joining method [36] using the Mega X software [37]. An ANI calculator (Average Nucleotide Identity) (http://enve-omics.ce.gatech.edu/ani/; accessed on 10 February 2022) [38,39] was used to compare *R. meliponini* D21B to all available *Rosenbergiella* genomes. Default parameters (genome fragments of 1000 bp window size and 200 bp step size) were used. Alignments were filtered based on 700 bp minimum length, 70% minimum identity, and 50 minimum alignments. In addition, the Type (Strain) Genome server (TYGS) analysis tool (http://ggdc.dsmz.de/; accessed on 14 August 2022) of the German Collection of Microorganisms and Cell Cultures (DSMZ) [40] was used to compare both the 16*S* rRNA gene and whole draft genome of *R. meliponini* D21B to those of *Rosenbergiella nectarea* 8N4, *R. epipactidis* 2.1A, *R. australiborealis* CdVSA20.1, and *R. collisarenosi* 8.8A.

Moreover, the whole genome Average Nucleotide Identity (ANI) score (http://enve-omics.ce.gatech.edu/ani/; accessed on 10 February 2022) was calculated between *R. meliponini* D21B and the currently recognized *Rosenbergiella* type strains. It is widely accepted that distinctive strains within a species share ANI scores of greater than 95% and that two strains that share ANI scores of less than 95% are distinctive species [38].

### 2.11. Physiological and Biochemical Characterization of R. meliponini D21B

Oxidase activity was determined using 1% *N*,*N*,*N*′,*N*′-tetramethyl-*p*-phenylenediamine dihydrochloride (Sigma-Aldrich, Schnelldorf, Germany) [41], and catalase activity was determined by adding hydrogen peroxide (3% *v*/*v*) onto an isolated bacterial colony [42]. EnteroPluri tests (Liofilchem srl, Roseto degli Abruzzi, Italy) were carried out according to the manufacturer’s instructions, with one significant variation: rather than incubating the tubes at 37 °C for 24 h, as recommended, the tubes were incubated for 2 days at 28 °C, owing to the poor growth of the tested *Rosenbergiella* strains at 37 °C.

In order to assess tolerance to osmotic stress, all *Rosenbergiella* strains were incubated in a salt-free LB medium (10 g/L peptone, 5 g/L yeast extract) with 0–80% *w*/*v* sucrose. Cell density was determined periodically by measuring the absorbance at 600 nm. After 11 days, 50 μL of culture was added to J agar to ascertain if viable cells were still present. Similarly, the salt tolerance was tested using 0–10% *w*/*v* sodium chloride in LB over the course of 11 d.

To test the capacity of *R. meliponini* D21B and *R. epipactidis* 2.1A to ferment carbohydrate sources other than those provided by the EnteroPluri tubes, 4 mL each of peptone/phenol red agar (20 g/L casein peptone, 10 g/L NaCl, 16 mg/L phenol red, 15 g/L agar) and a carbohydrate source in agar (20 g/L carbohydrate, 15 g/L agar) were mixed in a sterile test tube. The agar tubes were inoculated with the test bacterium. Carbohydrate sources assayed in this manner were fructose, galactose, mannose, ribose, lyxose (Aldrich, Taufkirchen, Germany), mannitol, sorbitol, lactose, maltose, sucrose, glycerol, trehalose (BLDpharm, Karlsruhe, Germany), and arabinogalactan (TCI, Zwijndrecht, Belgium), along with glucose (Merck, Taufkirchen, Germany) as a positive control.

### 2.12. Electron Microscopy of R. meliponini D21B

Laviad-Shitrit et al. [43] reported that *Rosenbergiella nectarea* cells were flagellated when grown in LB but non-flagellated when grown in LB supplemented with 10% sucrose. For comparison, we obtained electron micrographs of D21B that was grown at 28 °C, 140 rpm overnight under both conditions. Twenty microliters of the overnight cell suspension (at stationary phase of growth) was collected by centrifugation for 90 s at 10,000 rpm. The cells were fixed in glutaraldehyde (2% in phosphate-buffered saline pH 7.2, 1000 μL) and allowed to stand for 10 min. A 20 μL aliquot was loaded into an electron microscopy grid (EMC 1705), washed with doubly deionized water (5 × 8 s), and stained with uranyl acetate (1% in water). A Zeiss Auriga FIB-FESEM scanning electron microscope (Jena, Germany) was used in scanning transmission electron micrograph (STEM) mode to acquire the images.

### 2.13. Fatty Acid Methyl Ester (FAME) Profile of Hydrolyzed Lipids from R. meliponini D21B

The FAME profile of *R. nectarea* 8N4 has already been reported [44]. The FAME profile of *R. meliponini* D21B was analyzed by MIDI [45] at the DSMZ (Deutsche Sammlung von Mikroorganismen und Zellkulturen GmbH), Braunschweig, Germany.

Moreover, the FAME profiles of *Rosenbergiella* sp. D08K and *Rosenbergiella* sp. D15G, along with *R. epipactidis* 2.1A and *R. meliponini* D21B, were analyzed and compared qualitatively. Each *Rosenbergiella* strain was grown in NSLB supplemented with 10% sucrose (8 mL) overnight, and the cells were harvested by centrifugation (RCF 4162, 20 min). The methyl esters were obtained following the MIDI technical note [45]. Fatty acid methyl esters were analyzed by GC-MS (see Supplementary Materials).

### 2.14. Fatty Acid Trimethylsilyl Ester Profile of Hydrolyzed Lipids from R. meliponini D21B

All four *Rosenbergiella* strains were grown as described above, and cells were harvested and saponified following the MIDI protocol [45]. After saponification, the samples were acidified to pH 1 with HCl (6 M) and extracted with petroleum ether (3 mL). The petroleum ether extracts were dried over Na_2_SO_4_ and filtered, and the residues were evaporated to dryness under a stream of nitrogen. Samples were derivatized with *N*-methyl-*N*-trimethylsilyltrifluoroacetamide (MSTFA, 10 μL) for 1 h at 40 °C [46]. The fatty acid trimethylsilyl esters were analyzed by GC-MS (see Supplementary Materials).

### 2.15. Spent Liquid Medium Extractions for the Detection of Lipophilic Secondary Metabolites

Cells were grown in RYS broth (100 mL) for 6 days at (28 °C, 150 rpm) and harvested by centrifugation (RCF 4162, 20 min). The supernatant (pH 4.5–5) was acidified to pH 1 by the addition of HCl (32% *w*/*v*) and extracted with diethyl ether (50 mL). Emulsified layers were separated by mild centrifugation (RCF 738). The organic extracts were dried over Na_2_SO_4_ and filtered, and the solvent was removed by rotary evaporation. Prior to evaporation, a few drops of diethyl ether extracts were evaporated under a stream of nitrogen, derivatized with MSTFA, as previously described, dissolved in petroleum ether (1 mL), and analyzed by GC-MS (see Supplementary Materials).

### 2.16. Collection of Volatiles from R. meliponini D21B

*Solid phase microextraction (SPME). Rosenbergiella* cultures were grown on NSLB agar supplemented with sucrose (10% *w*/*v*). Volatiles were collected after 1, 3, and 6 days for 30 min using an SPME fiber (100 μm, polydimethylsiloxane coating, Restek, Bellefonte, PA, USA) that was inserted through a drilled hole at the side of the agar plate. The collected volatiles were analyzed by GC-MS desorbing the SPME fiber in the GC injection port (see Supplementary Materials).

*Closed loop stripping*. Five agar plates, each containing RYS broth (30 mL), were incubated at room temperature in a glass desiccator (3.0 L). After 3 days, the volatiles in the headspace of the desiccator were collected on charcoal filters using a closed loop stripping pump (2.0 V, DC06/18F pump, Fürgut GmbH, Tannheim, Germany) for 2 h [47]. The collected volatiles were eluted from the charcoal filters using ethyl acetate (3 × 30 μL), and the samples were analyzed by GC-MS (see Supplementary Materials).

### 2.17. Quantification of 2-Phenylethanol and 2-Phenylacetic Acid

The quantification of 2-phenylethanol and 2-phenylacetic acid production by *R. meliponini* D21B and *R. epipactidis* 2.1A is described in the Supplementary Materials.

### 2.18. Antibiotic Resistance of the Rosenbergiella Isolates

In order to investigate antibiotic resistance, *R. epipactidis* 2.1A, *R. meliponini* D21B, *R. meliponini* D08K, and *R. meliponini* D15G were cultivated in the presence of selected common antibiotics. One hundred microliter aliquots of bacterial cells grown overnight in RYS broth were added to 10 mL of RYS broth and mixed well. One hundred microliter aliquots of the inoculated broth were used to inoculate a 96-well plate, except for the cells in the first row of the plate, to which 75 μL of sterile RYS broth were added instead. The first row was mixed with 75 μL of antibiotic stock solution in RYS broth. Fifty microliters of the concentrated antibiotic/cell mixture was transferred to the adjacent well. This process was repeated until five 1:2 serial dilutions from the initial mixture were obtained. Fifty microliters of the final dilution in the series was discarded to maintain a constant volume.

The antibiotics assessed in this manner were ampicillin (initial concentration 1800 μg/mL), kanamycin (initial concentration 900 μg/mL), chloramphenicol (initial concentration 360 μg/mL), and novobiocin (initial concentration 3600 μg/mL). These concentrations were chosen so that a 1:1 dilution with inoculated culture medium would result in an antibiotic concentration ninefold higher than the recommended concentration to inhibit *E. coli* [48,49,50]. Thus, serial dilutions afforded the following antibiotic concentrations relative to the recommended working concentrations against *E. coli*: 9×, 3×, 1×, 0.3×, 0.1×, and 0.04×. Each antibiotic resistance test was assessed in triplicate. *E. coli* Top 10 was also assessed in this manner. The plates were incubated at 28 °C, 120 rpm for 3 days. The optical densities at 600 nm were measured using a Spectramax iD3 plate reader (Molecular Devices).

## 3. Results

### 3.1. 16S rRNA Sequence Analysis of the Isolates from T. carbonaria

*R. meliponini* D21B was isolated from a pollen pot of a *T. carbonaria* hive, while *Rosenbergiella* sp. D08K and *Rosenbergiella* sp. D15G were isolated from the hindgut of a dissected *T. carbonaria* bee.

The 16*S* rRNA gene of *R. meliponini* D21B was examined against other *Rosenbergiella* species. *R. meliponini* D21B exhibited 99.93% sequence identity with *R. epipactidis* 2.1A, 99.66% with *R. australiborealis* CdVSA 20.1, 99.80% with *R. collisarenosi* 8.8A, and 99.46% with *R. nectarea* 8N4 (Table S5), placing *R. meliponini* D21B within the genus *Rosenbergiella*.

*Rosenbergiella* sp. D08K and *Rosenbergiella* sp. D15G, isolated from the hindgut of the stingless bee, exhibited 99.27–99.85% 16*S* rRNA gene sequence identity with other *Rosenbergiella* strains. All three *Rosenbergiella* isolates from *T. carbonaria* contain the characteristic gene fragment (5′-GGTGTGAAATTAATACTTTCATG-3′), described as unique to *Rosenbergiella* [44].

**Figure 1:**
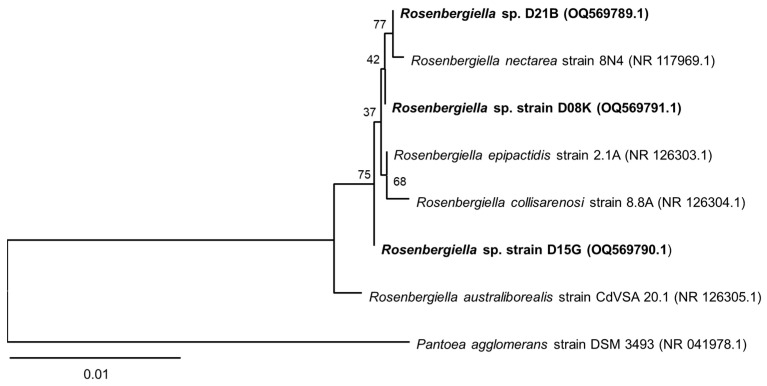
Neighbor-joining tree for the phylogenetic placement of the *Rosenbergiella* isolates from *T. carbonaria* based on 16*S* rRNA gene sequences. *R. meliponini* D21B strain clusters together with the type strains of *Rosenbergiella*, as well as *Rosenbergiella* sp. D08K and *Rosenbergiella* sp. D15G. *Pantoea agglomerans* DSM 3493 was used as the outgroup species. Bootstrap values calculated from 1000 replicates are indicated at branching nodes. Scale bar represents 0.01 substitutions per nucleotide position.

*R. australiborealis* CdVSA 20.1 occurs in a separate branch on the 16*S* rRNA phylogenetic tree (Figure 1). However, the 16*S* rRNA gene provides little bootstrap support (<75%) to distinguish between other type strains of the genus adequately [51]. Similarly, the 16*S* rRNA gene sequences of the three strains, *R. meliponini* D21B, *Rosenbergiella* sp. D08K and *Rosenbergiella* sp. D15G provide insufficient distinction from the four *Rosenbergiella*-type strains. Therefore, further investigations, such as the examination of other housekeeping genes and biochemical characterization, are necessary to distinguish the various *Rosenbergiella* species.

### 3.2. Genome of R. meliponini D21B

The genome of *R. meliponini* D21B was sequenced and assembled by Eurofins Genomics. The resulting draft genome consisted of 3,042,366 base pairs in 21 scaffolds (Table 1). The draft genome was annotated using the IMG Annotation Pipeline v.5.0.20. It contained 2924 protein-coding genes and 55 RNA-coding genes. Putative functions were predicted for 2521 of the proteins. Two thousand four hundred and ninety-four protein-coding genes were found within clusters of orthologous groups (COGs) (Table S6). The draft genome of *R. meliponini* D21B is accessible at the Integrated Microbial Genomes System database (img.igi.doe.gov) with the genome ID: 2901316999.

### 3.3. Phylogenetic Comparison of R. meliponini D21B and R. epipactidis 2.1A

**Figure 2:**
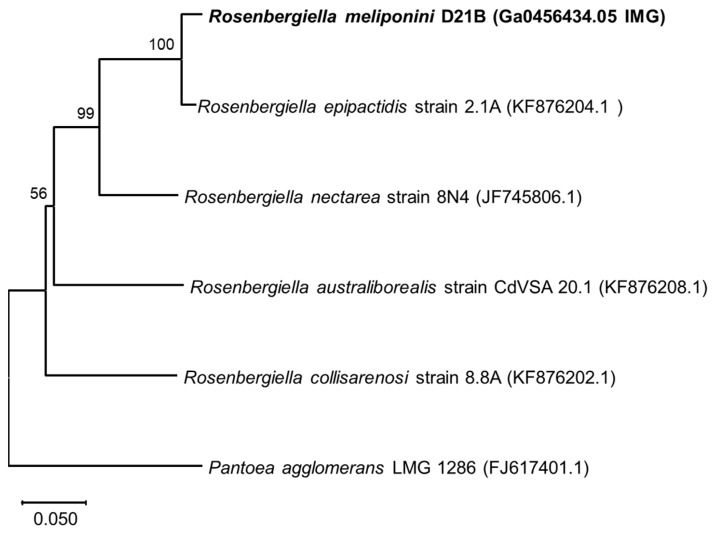
Neighbor-joining phylogenetic tree based on *gyrB* gene sequences. *R. meliponini* D21B and *R. epipactidis* 2.1A shared 97.26% sequence similarity. *R. meliponini* D21B exhibited 87.43%, 82.21%, and 81.19% sequence identity with *R. nectarea*, *R. australiborealis*, and *R. collisarenosi*, respectively. Bootstrap values > 50% calculated from 1000 replicates are indicated at branching nodes. Scale bar indicates 0.05 substitutions per nucleotide position.

**Figure 3:**
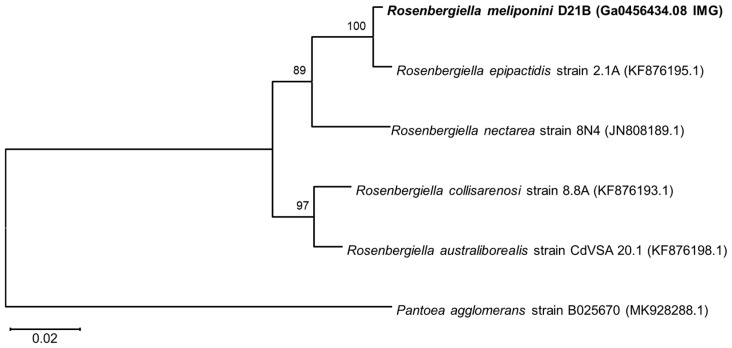
Neighbor-joining phylogenetic tree based on *atpD* gene sequences. *R. meliponini* D21B *atpD,* and *R. epipactidis* 2.1A *atpD* shared 99.19% sequence identity. With *R. nectarea*, *R. australiborealis* and *R. collisarenosi R. meliponini* D21B shared <96.08% sequence identity. Bootstrap values >50% calculated from 1000 replicates are indicated at branching nodes. Scale bar indicates 0.02 substitutions per nucleotide position.

**Figure 4:**
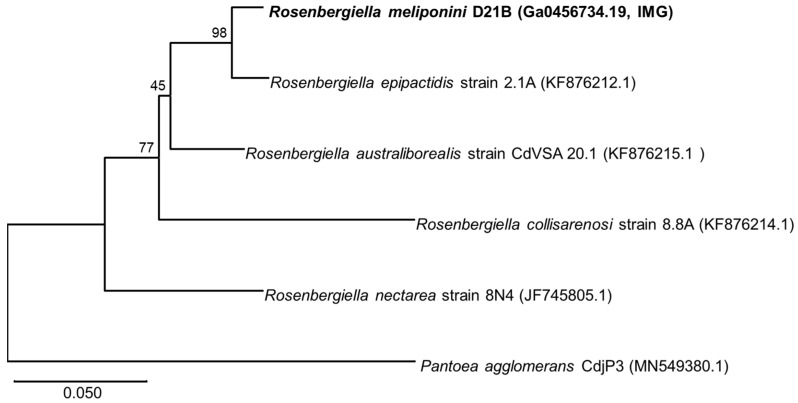
Neighbor-joining phylogenetic tree based on *rpoB* gene sequences. *R. meliponini* D21B *rpoB* and *R. epipactidis* 2.1A *rpoB* shared 97.43% sequence identity, whereas *R. meliponini* D21B *rpoB* shared less than 93.86% sequence identity with *rpoB* from *R. nectarea*, *R. australiborealis*, and *R. collisarenosi*. Bootstrap values > 45% calculated from 1000 replicates are indicated at branching nodes. Scale bar indicates 0.05 substitutions per nucleotide position.

The housekeeping genes *gyrB* (DNA gyrase subunit B), *rpoB* (RNA polymerase B’ subunit), and *atpD* (ATP synthase subunit β) were used for phylogenetic comparison with the sequences from *Rosenbergiella nectarea* 8N4 draft genome [43,51] as well as other *Rosenbergiella* genomes available at the NCBI genomes database (https://www.ncbi.nlm.nih.gov/genome/ accessed on 10 November 2020). *R. meliponini* D21B *gyrB* shared 81–97% sequence identity with *gyrB* of the other *Rosenbergiella* strains (see Figure 2, Table S5). The *aptD* gene of *R. meliponini* D21B exhibited 94–99% sequence identity with *atpD* of the other *Rosenbergiella* strains (Figure 3). The *rpoB* gene of *R. meliponini* D21B exhibited 86–97% sequence identity with *rpoB* of the other *Rosenbergiella* strains (Figure 4). More detailed phylogentetic trees considering additional latest reported *Rosenbergiella* strains [52] can be found in the Supplementary Materials (Figures S4–S7).

Phylogenetic analysis of the housekeeping genes *gyrB*, *atpD*, and *rpoB* revealed that *R. meliponini* D21B is most closely related to *R. epipactidis* 2.1A with >97.2% sequence similarity. Nevertheless, for all housekeeping genes analyzed, *R. meliponini* D21B and *R. epipactidis* 2.1A were clearly separated into individual branches of the neighbor-joining tree with strong bootstrap support ≥99% (Figure 2, Figure 3 and Figure 4).

Moreover, the Type Strain Genome Server (TYGS) analysis tool suggested that *R. meliponini* D21B is a novel species (Table 2). The dDDH value derived from the TYGS formula d4 (GGDC formula 2) is the sum of all identities found in high-scoring segment pairs (HSPs; genomic regions from both genomes with a high degree of matching) divided by the overall HSP length. The dDDH value for the d4 formula of *R. meliponini* D21B and *R. epipactidis* 2.1A was 61.4%, providing justification for a new species using the 70% threshold [53]. In TYGS, the d4 calculation is preferred in the case of a comparison of draft genomes because this value is independent of the genome length.

In addition, the Average Nucleotide Identity (ANI) score of *R. meliponini* D21B and *R. epipactidis* 2.1A revealed an ANI score of 94.85, indicating *R. meliponini* D21B (NG 299429) is genetically distinct enough to be classified as a novel species [38] (Table S4). However, ANI estimations can be less accurate for incomplete draft genomes [54].

A comparison of the housekeeping genes of *R. meliponini* D21B with those of the recently reported *R. metrosideri* strain JB07 indicated that both strains are closely related (Figures S4–S7) [52]. However, there were substantial differences in the biochemical properties between *R. metrosideri* strain JB07 and *R. meliponini* D21B (Table S8) that clearly defined them as different species.

### 3.4. Analysis of Secondary Metabolite Biosynthetic Gene Clusters from R. meliponini D21B

In order to assess the potential of *R. meliponini* D21B to produce secondary metabolites, its genome was subjected to antiSMASH analysis [55]. Only five secondary metabolite gene clusters were identified (Figure S9). A putative carotenoid biosynthetic gene cluster (scaffold 18, locus 104,611–111,500) exhibited 100% similarity to that of *Pantoea ananatis* PA13 [56] and was highly conserved across all four other sequenced *Rosenbergiella* species. *R. meliponini* D21B contained biosynthetic gene clusters coding for enzymes that produce siderophores. A biosynthetic gene cluster (scaffold 8, locus 74,680–83,302) exhibited 100% similarity to the desferrioxamine E biosynthetic gene cluster from *Pantoea agglomerans* [57,58]. Again, these genes were highly conserved in all sequenced *Rosenbergiella* species. Moreover, all sequenced *Rosenbergiella* genomes, including *R. meliponini* D21B, contained genes (scaffold 6, locus 67,757–110,721) similar to those coding for a putative enterobactin-like siderophore biosynthetic gene cluster, although with low similarity (12–45%) [59]. A biosynthetic gene cluster (scaffold 11, locus 61,112–76,420) was also conserved in all *Rosenbergiella* genomes and was predicted to encode for a pyrroloquinoline (PQQ) redox cofactor [60]. The gene cluster (scaffold 6, locus 116,225–170,636) codes for enzymes putatively involved in the formation of a mixed non-ribosomal peptide synthetase polyketide synthase product. However, two of the adenylation domains of this cluster were annotated as inactive, so that this gene cluster may have lost its function. Nevertheless, this biosynthetic gene cluster appears to be unique among *Rosenbergiella* genomes, and there are also no known closely related biosynthetic gene clusters from other microorganisms. Because the genome of *R. nectarea* 8N4 contains a gene that encodes for an S-type pyocin protein (Table S11), we screened the other *Rosenbergiella* genomes for it. However, this gene was not observed in any other type strains, including *R. meliponini* D21B. A putative gene that may encode for colicin V (cvpA) was detected in the *R. meliponini* D21B genome (Table S11). Colicin is a bacteriocin commonly produced by *E. coli* [61]. However, several other genes that encode for accessory proteins necessary for immunity and/or resistance to colicin V were not identified in *R. meliponini* D21B, although they are present in *R. australiborealis* CdVSA20.1 and *R. collisarenosi* 8.8A. This may indicate that the colicin gene cluster has lost some components in *R. meliponini* D21B and is no longer functional.

### 3.5. Physiological and Biochemical Characterization of R. meliponini D21B

*R. meliponini* D21B was oxidase negative and catalase positive. It was a facultative anaerobe. *R. meliponini* D21B exhibited enhanced growth in media supplemented with 10–20% sucrose relative to a corresponding sucrose-free medium, and growth was observable at sucrose concentrations of up to 50%. However, when *R. meliponini* D21B in 80% sucrose was plated onto J agar after 5 days of incubation, it grew again, demonstrating that cells remained viable at high concentrations of sucrose. *R. meliponini* D21B tolerated up to 8% NaCl. Again, after exposure to 10% NaCl for 5 days and no visible cell growth (OD_600_), viable cells could be recovered after switching the medium. These growth characteristics are similar to those described for other *Rosenbergiella* species (Table S8) [44,51].

The EnteroPluri tube results were largely the same across the four strains tested, with the exception of the urease and catalase tests. These were both negative for *R. epipactidis* 2.1A but positive (albeit weakly in some cases) for *R. meliponini* D21B, *Rosenbergiella* sp. D08K, and *Rosenbergiella* sp. D15G. No differences were observed between *R. epipactidis* 2.1A and *R. meliponini* D21B in the metabolism of carbohydrates other than those assayed by the EnteroPluri tubes (Table S12).

### 3.6. Minimal Growth Requirements of Rosenbergiella

**Figure 5:**
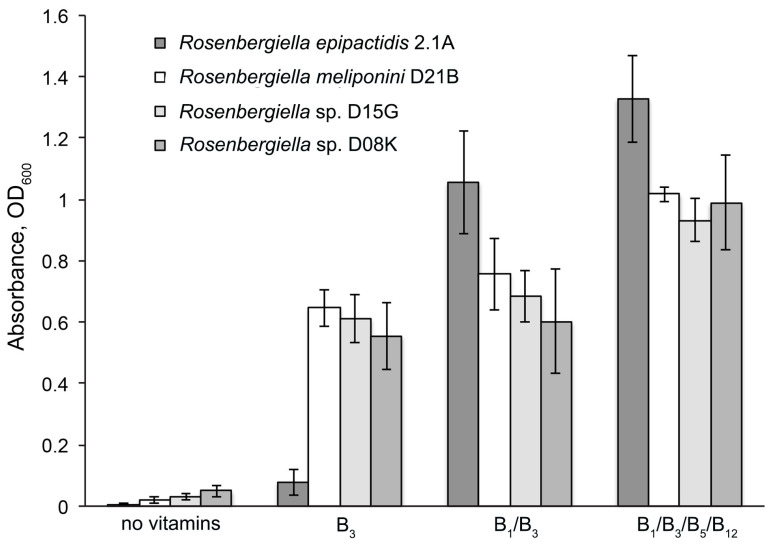
Vitamin B requirements of Rosenbergiella strains: *R. epipactidis* 2.1A required vitamin B_1_ and B_3_ for growth, while *R. meliponini* D21B, *Rosenbergiella* sp. D15G, and *Rosenbergiella* sp. D08K required only vitamin B_3_ to grow in a minimal medium. No strain required vitamin B_5_ or B_12_, although cell cultures sometimes attained higher densities when “non-essential” vitamins were supplied.

*R. meliponini* D21B, *Rosenbergiella* sp. D08K, *Rosenbergiella* sp. D15G, and *R. epipactidis* 2.1A, grow in a range of complex media such as yeast extract sucrose medium [62], J medium [63], LB medium, and SD medium [64]. Growth was less luxuriant on SD agar and LB agar than it was on agars supplemented with yeast extract and a suitable carbohydrate source such as glucose or sucrose. All *Rosenbergiella* tested here grew in the minimal medium adapted from Thrunheer et al. [29] with supplemented B-group vitamins [30]. Complete gene clusters involved in vitamin biosynthesis were identified in the draft genome of *R. meliponini* D21B for 4-aminobenzoic acid (folate precursor), biotin, and pyridoxine. However, for thiamine, nicotinic acid, pantothenic acid, and cobalamin it was less clear if *Rosenbergiella* strains contained the complete set of genes for their synthesis. These four vitamins were, therefore, selectively removed from the culture medium in order to establish the minimum vitamin requirements of the *Rosenbergiella* strains. Consistent with our bioinformatic analysis, all *Rosenbergiella* strains tested here grew in a minimal media without supplementation of 4-aminobenzoic acid, biotin, and pyridoxine. Selective removal of thiamine, nicotinic acid, pantothenic acid, and cobalamin from the growth medium revealed that nicotinic acid was the only essential vitamin for all organisms, and *R. epipactidis* 2.1A also grew poorly in the absence of thiamine (Figure 5, Table S14). This would imply that the tested strains can synthesize most of the other vitamins and cofactors, even those for which full biosynthetic gene clusters were not clearly identified in the draft genome. Cobalamin may be an exception to this, as the presence of a gene encoding a vitamin B_12_-independent methyltransferase in the genome of *R. meliponini* D21B (Table S23) may indicate that cobalamin is not an essential cofactor.

All *Rosenbergiella* genomes examined contained all seven genes of the Shikimate pathway for the production of aromatic amino acids and folates (Table S25) [65]. The presence of biosynthetic pathways for all amino acids can be inferred from the ability of all four strains (*R. meliponini* D21B, *Rosenbergiella* sp. D08K, *Rosenbergiella* sp. D15G, and *R. epipactidis* 2.1A) to grow in a minimal medium with ammonium chloride as the sole nitrogen source, although the exact organization of these amino acid synthesis pathways remains to be studied in detail [66].

### 3.7. Electron Microscopy of R. meliponini D21B

**Figure 6:**
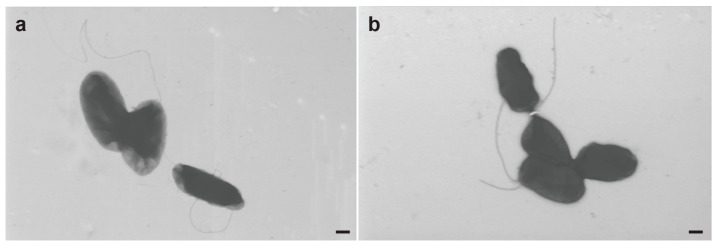
Electron microscopy pictures of *R. meliponini* D21B. (**a**) Cells were grown in a salt-free LB medium supplemented with 10% sucrose. (**b**) Cells were grown without sucrose in a salt-free LB medium. Scale bars indicate a length of 200 nm.

The size of *R. meliponini* D21B cells varied from 0.3–0.9 μm in width and 0.5–1.8 μm in length (Figure 6). Because it has been reported that *R. nectarea* 8N4 flagellum development was suppressed in the presence of sucrose [43], *R. meliponini* D21B was grown for 16 h in NSLB medium, both with and without sucrose (10% *w*/*v*). Cells from each growth medium were examined for the presence of flagella by electron microscopy. *R. meliponini* D21B reached >30 million colony-forming units per microliter and formed aggregates in both media. Flagella were observed in about 50% of cells, irrespective of the presence or absence of sucrose in the medium.

### 3.8. Fatty Acid Profile of Hydrolyzed Lipids

The major cellular fatty acids of *R. meliponini* D21B (>5% according to DSMZ MIDI GC-analysis service) [45] were myristic acid (8.27%), summed feature 2 (C14:0 3OH/C16:1 iso I, 6.53%), stearic acid (35.30%), C_17:0 cyclo_ (21.72%), and summed feature 8 (C18:1 ω6c/ω7c, 14.94%). Further investigation of the hydrolyzed fatty acids using derivatization with MSTFA and GC-MS analysis revealed that summed feature 2 was 3-hydroxymyristic acid (C14:0 3OH) and not a C16 unsaturated fatty acid and that the fatty acid compositions for *R. meliponini* D21B, *Rosenbergiella* sp. D08K, *Rosenbergiella* sp. D15G, and *R. epipactidis* 2.1A were qualitatively similar (see Supplementary Materials). These results revealed a sharp distinction within the genus *Rosenbergiella*. *R. nectarea* 8N4 shared with *R. epipactidis* 2.1A and the stingless bee isolates palmitic acid, a C17 cyclopropyl fatty acid and a C18 unsaturated fatty acid, but also has a “summed feature 3” (C16:1 ω7c and/or iso-C15:0 2-OH) [44]. The reported fatty acid profile of *R. nectarea* 8N4, which was limited to fatty acids >10% of the total fatty acid composition, does not include 3-hydroxymyristic acid or a summed feature that might correspond to this fatty acid.

### 3.9. Production of 2-Phenylethanol by the Rosenbergiella Isolates from T. carbonaria

**Figure 7:**
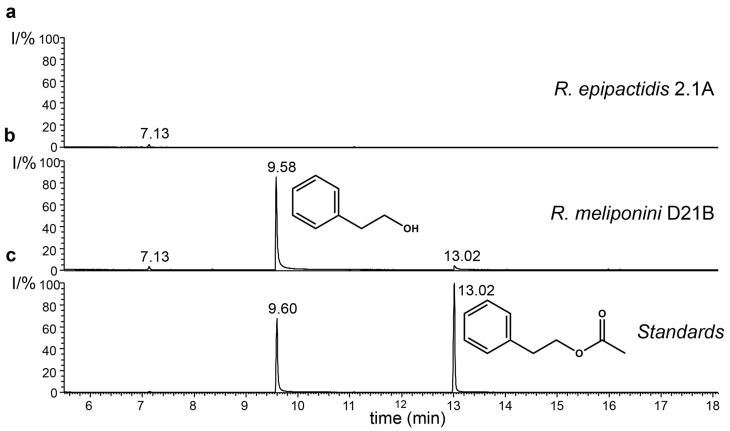
Comparison of 2-phenylethanol and 2-phenylethyl acetate production by *R. meliponini* D21B and *R. epipactidis* 2.1A. (**a**) Total ion current chromatogram of SPME collected volatiles from *R. epipactidis* 2.1A. (**b**) Total ion current chromatogram of SPME collected volatiles from *R. meliponini* D21B. (**c**) Total ion current chromatogram (TIC) of SPME collected 2-phenylethanol and 2-phenylethyl acetate standards.

Analysis of the headspace of *R. meliponini* D21B, *Rosenbergiella* sp. D08K and *Rosenbergiella* sp. D15G by GC-MS revealed that all *Rosenbergiella* isolated from *T. carbonaria* produced large quantities of 2-phenylethanol (Figure 7). Traces of 2-phenylethyl acetate also became detectable from day 3 onwards. In contrast, *R. epipactidis* 2.1A did not synthesize 2-phenylethanol or any other volatile compounds detectable by SPME or closed-loop stripping. The 2-phenylethanol biosynthetic pathway has been studied in *Proteus mirabilis* [67]. All genes that encode necessary components in 2-phenylethanol biosynthesis pathway were conserved in *R. meliponini* D21B and *R. epipactidis* 2.1A. In the case of α-keto acid decarboxylase (WP_012367760.1) and pyridoxal-dependent decarboxylase (WP_017628132.1), the level of conservation was low (Tables S25 and S26). Nevertheless, the presence of these enzymes, or those with similar activity, can be inferred by the production of 2-phenylethanol in *R. meliponini* D21B.

### 3.10. Analysis of spent medium of R. epipactidis 2.1A and R. meliponini D21B

**Figure 8:**
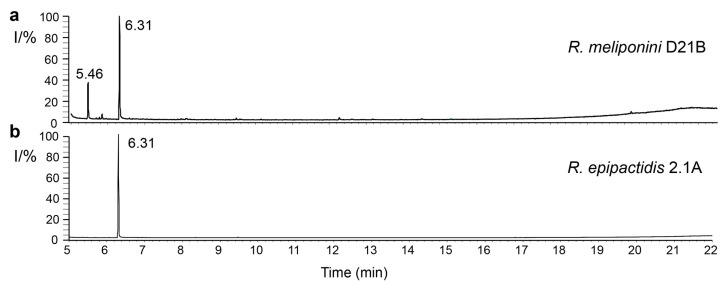
Comparison of total ion current chromatograms of (**a**) *R. meliponini* D21B and (**b**) *R. epipactidis* 2.1A spent medium of ethyl acetate extracts after derivatization with MSTFA. The two major observable products were identified as 2-phenylethanol (RT 5.46 min) and 2-phenylacetic acid (RT 6.31 min). See Supplementary Materials for EI-MS data.

Ethyl acetate extracts of 6-day-old spent medium from *R. meliponini* D21B and *R. epipactidis* 2.1A were compared by GC-MS after derivatization with MSTFA. As expected from the analysis of the volatile constituents, all three strains isolated from *T. carbonaria* produced 2-phenylethanol. 2-Phenylacetic acid was additionally detected in all *Rosen-bergiella* examined, including *R. epipactidis* 2.1A (Figure 8).

### 3.11. Quantification of 2-Phenylethanol and 2-Phenylacetic Acid Production by Rosenbergiella

*R. epipactidis* 2.1A produced 0.19 g/L of 2-phenylacetic acid and no observable 2-phenylethanol, after incubation for 6 days in RYS broth at 28 °C. Under the same conditions, *R. meliponini* D21B produced only 0.03 g/L of 2-phenylacetic acid, and 0.18 g/L of 2-phenylethanol (Table S3).

### 3.12. Antibiotic Resistance of Rosenbergiella Strains

The genome of *R. meliponini* D21B comprised genes coding for three multidrug efflux pumps (Table S27). Thus, we tested to which extent the *Rosenbergiella* isolates were resistant to representative antibiotics, ampicillin, chloramphenicol, kanamycin, and novobiocin (Table S7). *R. meliponini* D21B tolerated ampicillin and chloramphenicol 9–10 times better than *E. coli* Top10 and kanamycin three times better, whereas *E. coli* Top 10 exhibited three times higher resistance to novobiocin than R. *meliponini* D21B. Antibiotic resistance of *Rosenbergiella* sp. D08K, *Rosenbergiella* sp. D15G, and *R. epipactidis* 2.1A were not necessarily the same as that of *R. meliponini* D21B, although all were qualitatively similar when compared to *E. coli* Top10, being more sensitive to novobiocin, but as resistant or more resistant than *E. coli* Top10 to ampicillin, kanamycin, and chloramphenicol.

## 4. Discussion

The genus *Rosenbergiella* was first described in 2013 when Halpern et al. isolated a novel Enterobacterium *R. nectarea* 8N4 from the nectar of both *Amygdalus communis* (almond) and *Citrus paradisi* (grapefruit) in Israel [44]. The next year, Lenaerts and co-workers added *R. australiborealis* CdVSA20.1, *R. collisarenosi* 8.8A, and *R. epipactidis* 2.1A, isolated from nectar samples from plants growing in France, Belgium, Spain, and South Africa [51]. No new species were described until 2023, when Álvarez-Pérez et al. added *Rosenbergiella gaditana* strain S61 and *Rosenbergiella metrosideri* strain JB07 to the list [52]. Since the description of the first four species by 2014, *Rosenbergiella* strains have been identified in pollen samples [68,69]. Furthermore, Manirajan et al. [69] noted that *Rosenbergiella* were more closely associated with insect-pollinated plants than with wind-pollinated plants, an association that has been echoed by the detection of *Rosenbergiella* through metagenomic studies of hive and nest samples taken from honeybees (both *Apis mellifera* and *Apis cerana*) [70,71], the alfalfa leafcutter bee *Megachile rotundata* [72], the small carpenter bees (genus *Ceratina*) [73], stingless bees from Australia [70], and bumblebees in Europe and China [1,74]. Interestingly, *Rosenbergiella* strains were not identified in the Eastern American bumblebee *Bombus impatiens* [75]. None of these culture-independent studies reported the isolation or characterization of *Rosenbergiella* from bees. However, in 2023, Álvarez-Pérez et al. described the isolation of a strain of *R. epipactidis* from the crop of the European honeybee *Apis mellifera*, along with the isolation of a strain of *R. nectarea* from the mouth of a honeybee, and another strain of *R. epipactidis* from the gut of a bumblebee [52].

Here, we have isolated three *Rosenbergiella* strains for the first time from a *T. carbonaria* stingless beehive, from both pollen pots and the lower digestive tract of a worker bee. Because *Rosenbergiella* was isolated multiple times from *T. carbonaria,* it is conceivable that these microorganisms play an important role in the ecology of *T. carbonaria*. This is in line with previous observations that identified *Rosenbergiella* in metagenomics screens of bees [1,70,71,72,73,74], as well as the isolation by Álvarez-Pérez et al. from honeybees and a bumblebee [52]. Moreover, *Rosenbergiella* has previously been observed more frequently in flowers pollinated by bees [68,69]. Thus, bees such as *T. carbonaria* likely acquire or distribute *Rosenbergiella* strains while foraging. The relationships between plants, pollinating insects, and *Rosenbergiella* are still largely unknown, although there is some evidence of mutualism between *Rosenbergiella* and bees. Most notably, Pozo et al. [76] observed that adding various yeasts and/or bacteria to food sources of captive *Bombus terrestris* resulted in a positive impact on nest development, with *R. nectarea* being one of the most beneficial organisms they tested. *R. meliponini* D21B from the pollen pots of *T. carbonaria* constitutes a new member of the little investigated, relatively newly discovered *Rosenbergiella* genus because it is phylogenetically and physiologically different from its most close relative, *R. epipactidis* 2.1A. A comparison of both genomes using ANI [39] and TYGS [40] analysis indicated a new species (Table S4).

Both *R. meliponini* D21B and *R. epipactidis* 2.1A metabolized nutrients in a similar way apart from urease activity and the utilization of citrate (Table S8). However, citrate utilization is variable even within *R. epipactidis* [51]. *R. meliponini* D21B tolerated high temperatures better than *R. epipactidis* 2.1A (Table S8). *R. meliponini* D21B had a unique NRPS/PKS gene cluster that was not present in *R. epipactidis* 2.1A. Unlike *R. epipactidis* 2.1A, *R. meliponini* D21B grew well without supplementation of thiamine to the minimal medium (Figure 5). Moreover, *R. meliponini* D21B produced large amounts of 2-phenylethanol as well as 2-phenylacetic acid, as do the other *Rosenbergiella* isolates from *T. carbonaria*. In contrast, *R. epipactidis* 2.1A produced only 2-phenylacetic acid but not 2-phenylethanol (see Figure 7 and Figure 8). A summary of the major differences between *R. meliponini* D21B and *R. epipactidis* 2.1A can be found in Table 3.

The draft genome of *R. meliponini* D21B did not reveal full biosynthetic clusters for cobalamin, pantothenic acid, and thiamine biosynthesis, but the bacterium grew in the absence of these vitamins, indicating that *R. meliponini* D21B can either synthesize these vitamins or does not require some of them. It is highly unlikely that *Rosenbergiella* can grow independently of pantothenic acid or thiamine, considering how crucial these cofactors are for a multitude of core biochemical processes [77,78,79], although the presence of a vitamin B_12_-independent methyl transferase (Table S23) may mean that cobalamin is not an essential cofactor for *Rosenbergiella*.

*R. meliponini* D21B—based on AntiSMASH analysis [55]—only comprised a few secondary metabolite biosynthetic gene clusters. In particular, there was a gene cluster for carotenoid biosynthesis and two putative gene clusters for the production of siderophores. Since the iron ion content of both pollen and honey can vary considerably [80,81,82], the efficient uptake of iron ions by siderophores will be essential for *Rosenbergiella* strains to survive in their natural habitat, explaining the presence of two siderophore producing gene clusters.

### 4.1. Possible Symbiotic Benefits of Rosenbergiella for Bees

*Rosenbergiella* may serve as mutualistic symbionts in bees and other pollinators in one or more of the following manners. Although our genome analysis and *in vitro* biochemical characterisation suggests some potential benefits for their host, future experiments are needed to address the potential role of *Rosenbergiella* for their stingless bee host.

### 4.2. Amino Acid and Vitamin Synthesis

All *Rosenbergiella* strains examined in this study grew in a minimal medium with ammonium chloride as the sole nitrogen source. They must therefore be able to synthesize all proteinogenic amino acids. They could, therefore, conceivably supply amino acids to other microbial symbionts in the lower gut, as does *Snodgrassella* in honey bees [8]. The presence of *R. meliponini* D21B in the pollen pots may mean that it plays a role in augmenting the amino acid profile of the bee bread. While pollen is generally high in protein [82,83], eucalyptus pollen, which constitutes a major part of the *T. carbonaria* diet [84], can be low in isoleucine [83].

The vitamin requirements for insects are generally not well established. However, the optimum level of pyridoxine (vitamin B_6_) for brood development in honeybees (*Apis mellifera*) reared on an artificial diet is 4–8 milligrams per kilogram of food [85]. This is somewhat higher than the concentrations of 2–7 mg/kg in bee bread reported by Denisow and Denisow-Pietrzyk [82], and Ciulu et al. [86] reported negligible quantities of pyridoxine per kilogram of honey. The presence of organisms that can synthesize vitamin B_6_ in fermented bee pollen could potentially result in a higher quality food source than a bee bread sample in which vitamin B_6_-dependent microbes are the predominant fermentative microorganisms. Vitamin B_6_ synthesized in the hindgut might also conceivably be available to hosts. Bees may also benefit from microbial biosynthesis of vitamins for which the bees’ minimal requirements are not yet well established.

### 4.3. Digestion of Food

Not all carbohydrates in pollen can be digested by bees. Pectin and cellulose are both present in plant cell walls, including pollen grains, and need to be broken down in order for the bee to digest pollen [87]. Pectin has even been identified to be toxic to honeybees (*Apis mellifera*) [87,88]. Therefore, microorganisms that can degrade pectin perform an important service to bees. Pectin-degrading enzymes were identified in the draft genome of *R. nectarea* 8N4 (Table S28), though interestingly, not in any other species sequenced to date, including *R. meliponini* D21B.

### 4.4. Ecological Role of Secondary Metabolites

All *Rosenbergiella* isolates from *T. carbonaria* produced both 2-phenylethanol and 2-phenylacetic acid in ca. 0.2 mg/mL and 0.03 mg/mL, respectively. Both compounds are most likely produced from 2-phenylacetaldehyde that originates from transamination and decarboxylation of phenylalanine (Ehrlich pathway) [89]. 2-Phenylacetic acid and 2-phenylethanol have pleasant odors. In particular, 2-phenylethanol exhibits a characteristic rose smell and occurs in a variety of plants [90]. It is also produced by some microorganisms, such as *Candida albicans* [91], *Erwinia carotovora* [92], *Microbacteria* [93], and *Brevibacteria* [92,94,95]. The characteristic smell of these secondary metabolites may play an ecological role. For example, stingless bees may be attracted to these chemicals, or they may play a role in helping bees identify and locate flowers for foraging. Additionally, 2-phenylethanol and 2-phenylacetic acid are both reported to exhibit antimicrobial activities [96,97,98,99,100]. Thus, the overproduction of 2-phenylethanol and 2-phenylacetic acid by *Rosenbergiella* isolates may help to protect *T. carbonaria* against pathogens.

## 5. Conclusions

*Rosenbergiella* appears to be associated with the Australian stingless bee *T. carbonaria*. *R. meliponini* D21B from *T. carbonaria* constitutes a new species of the so far little-studied *Rosenbergiella* genus. *R. meliponini* D21B is most closely related to *R. epipactidis* 2.1A. However, *R. meliponini* D21B not only exhibits clear phylogenetic differences but also some interesting physiological differences, such as higher thermal tolerance, the ability to grow without thiamine supplementation to the growth medium, and the release of 2-phenylethanol and 2-phenylethyl acetate. Some of these features may be beneficial for the stingless bee host. Generally, the ability of *Rosenbergiella* to synthesize all essential amino acids and most B-group vitamins may provide a fitness gain for insect hosts, which should be addressed in future experiments.

## Figures and Tables

**Table 1:** Genome characteristics of *R. meliponini* D21B.

	Number	% of Total
Total number of bases	3,042,366	100.00%
Number of coding bases	2,695,972	88.61%
G/C content	1,433,972	47.13%
NG50	299,429	
L50	4	
DNA scaffolds	21	100.00%
Genes (total number)	3023	100.00%
Protein coding genes	2924	96.73%
Regulatory and miscellaneous features	44	1.46%
RNA genes	55	1.82%
rRNA genes	5	0.17%
5*S* rRNA	3	0.10%
16*S* rRNA	1	0.03%
23*S* rRNA	1	0.03%
tRNA genes	47	1.55%
other RNA genes	3	0.10%
Protein coding genes with predicted function	2521	83.39%
Protein without function prediction	403	13.33%
Protein coding genes with enzymes	952	31.49%
Protein coding genes connected to KEGG pathways	1085	35.89%
Protein coding genes not connected to KEGG pathways	1839	60.83%
Protein coding genes connected to KEGG orthology (KO)	2001	66.19%
Protein coding genes not connected to KEGG orthology (KO)	923	30.53%
Protein coding genes connected to MetaCyc pathways	795	26.30%
Protein coding genes not connected to MetaCyc pathways	2129	70.43%
Protein coding genes with COGs3	2494	82.50%
with Pfam3	2564	84.82%
with TIGRfam3	1249	41.32%
with SMART	560	18.52%
with SUPERFam	2409	79.69%
with CATH FunFam	2080	68.81%
in internal clusters	539	17.83%

**Table 2:** Digital DNA-DNA hybridization (dDDH) values of *R. meliponini* D21B against sequenced *Rosenbergiella* strains calculated by TYGS (https://tygs.dsmz.de/) [40]. Legend: C. I.: confidence intervals, formula d0 (also known as Genome-to-Genome Distance Calculator (GGDC) formula 1): length of all high-scoring segment pairs (HSPs) divided by total genome length. Formula d4 (GGDC formula 2): sum of all identities found in HSPs divided by overall HSP length. Formula d6 (GGDC formula 3): sum of all identities found in HSPs divided by total genome length.

Strain	dDDH (d0, in %)	C.I. (d0, in %)	dDDH (d4, in %)	C.I. (d4, in %)	dDDH (d6, in %)	C.I. (d6, in %)	G + C Content Difference (in %)
*Rosenbergiella epipactidis* 2.1A	78.8	[74.8–82.3]	61.4	[58.6–64.2]	78	[74.6–81.1]	0.46
*Rosenbergiella nectarea* 8N4	73.9	[69.9–77.5]	31.1	[28.7–33.6]	61.6	[58.3–64.8]	0.31
*Rosenbergiella collisarenosi* 8.8A	51.3	[47.9–54.8]	20.7	[18.5–23.1]	39.8	[36.9–42.9]	1.04
*Rosenbergiella australiborealis* CdVSA20.1	47	[43.6–50.4]	20.2	[18.0–22.6]	37	[34.1–40.1]	1.82
*Pantoea cypripedii* LMG 2657	13.2	[10.5–16.5]	20	[17.8–22.4]	13.6	[11.2–16.3]	6.92

**Table 3:** Major physiological, biochemical, and genetic differences between *Rosenbergiella meliponini* D21B and its nearest relative, *R. epipactidis* 2.1A.

Characteristic	*R. meliponini* D21B1	*R. epipactidis* 2.1A
Urease activity	Yes	No
Utilization of citrate	Yes	No
Growth at 37 °C	Poor	No
NRPS/PKS gene cluster	Present	Absent
Production of 2-phenylethanol	Yes	No
Thiamine dependency	Independent	Dependent
Pyocin encoding genes	No	Yes
Hemolysin encoding genes	No	Yes

## Data Availability

The draft genome of *Rosenbergiella meliponini* D21B is available at IMG: Project ID: Gp0509688, Analysis ID: Ga0456434. The 16*S* rDNA sequences of the *Rosenbergiella* isolates are available at NCBI: OQ569789, OQ569790, OQ569791. *Rosenbergiella meliponini* D21B was deposited at the Belgian Co-Ordinated Collections of Micro-Organisms (BCCM) and National Collection of Industrial, Food and Marine Bacteria (NCIMB), and has the strain numbers LMG 32782 and NCIMB 15457, respectively. Additional data will be available from the authors upon request.

## Supplementary Materials

The following supporting information can be downloaded at: https://www.mdpi.com/article/10.3390/microorganisms11041005/s1. Cultivation and genetic analysis of Rosenbergiella meliponini D21B.
