# Supplementary material for: Rosenbergiella meliponini D21B Isolated from Pollen Pots of the Australian Stingless Bee Tetragonula carbonaria
Source: Microorganisms. 2023 Apr 12;11(4):1005. doi: 10.3390/microorganisms11041005 (PMC10142583; doi:10.3390/microorganisms11041005)
Supplement: Supplementary file 1 [file microorganisms-11-01005-s001.zip › microorganisms-2313761-supplementary.pdf]

Rosenbergiella meliponini D21B isolated from pollen pots of the Australian stingless bee *Tetragonula carbonaria*

Anthony J. Farlow[1], Darshani B. Rupasinghe[1], Khalid M. Naji[1], Robert J. Capon[2], and Dieter Spiteller[1*]

Table of contents

Cultivation of *Rosenbergiella* strains **3**

Gas chromatography-mass spectrometry (GC-MS) **4**

GC-MS program for the analysis of volatile organic compounds collected by SPME 4

GC-MS program for ethyl acetate extracts of spent *Rosenbergiella* medium 4

Analysis of fatty acid methyl esters from *Rosenbergiella* strains by GC-MS 4

Fatty acid trimethylsilyl esters from lipid hydrolysates of *Rosenbergiella* isolates and *R. epipactidis* 2.1A 7

Compounds identified by GC-MS in ethyl acetate extracts of spent medium of *R. meliponini* D21B 8

GC-MS analysis of volatile organic compounds released by the *Rosenbergiella* isolates and *R. epipactidis* 2.1A 9

Phylogenetic analysis of 16*S* rDNA and house-keeping genes of *Rosenbergiella* isolates from *T. carbonaria* **12**

Detailed neighbour joining phylogenetic trees of *Rosenbergiella* strains 12

ANI scores 17

Characteristics of the draft genome of *R. meliponini* D21B **19**

Secondary metabolite biosynthetic gene clusters from *R. meliponini* D21B **23**

Amino acid biosynthesis by *Rosenbergiella* strains 28

Production of vitamins by *Rosenbergiella* strains 28

Vitamin B_1 (thiamine) 30

Vitamin B_2 (Riboflavin) 32

Vitamin B_3 (Niacin) 33

Vitamin B_5 (Pantothenic acid) 35

Vitamin B_6 (pyridoxine) 36

Vitamin B_7 (biotin) 37

Vitamin B_9 (folate) 39

Vitamin B_{10} (4-aminobenzoic acid) 40

Vitamin B_{12} (cobalamin) 41

Biosynthesis of 2-phenylethanol **44**

Amino acid deaminase (AAD) pathway 44

The Ehrlich pathway 44

Aromatic amino acid decarboxylase (AADC) pathway 44

Resistance to environmental challenges **49**

Antibiotic resistance 49

Genes associated with virulence of *R. meliponini* D21B 51

Acid stress responses 53

Osmotic stress 54

Oxidative stress responses 57

References 60

Cultivation of *Rosenbergiella* strains

Solid medium for agar plates was supplemented with 15 g/l agar (Kobe).
Double distilled water was used to prepare the media.

J broth [1]
Yeast extract (15 g/l), casein tryptone (5 g/l), K_2HPO_4 (3 g/l), glucose (2 g/l, autoclaved separately). pH was adjusted to 7.3-7.5 using 2 N HCl.

Lysogeny broth Luria (LB Luria) [2]
Casein tryptone (10 g/l), yeast extract (5 g/l), sodium chloride (0.5 g/l).

No-Salt lysogeny broth (NSLB) [3]
Casein tryptone (10 g/l), yeast extract (5 g/l).

Yeast extract sucrose (YES) broth [4]
Sucrose (150 g/l), yeast extract (20 g/l), $MgSO_4 \cdot 7H_2O$ (0.5 g/l), $ZnSO_4 \cdot 7H_2O$ (10 mg/l), $CuSO_4 \cdot 5H_2O$ (5 mg/l).

***Rosenbergiella* yeast-sucrose (RYS) broth**
Sucrose (50 g/l), yeast extract (24 g/l), $MgSO_4 \cdot 7H_2O$ (0.5 g/l), $ZnSO_4 \cdot 7H_2O$ (10 mg/l), $CuSO_4 \cdot 5H_2O$ (5 mg/l).
This medium is based on the yeast extract sucrose medium described above, and was ideal for *Rosenbergiella* cultivation.

Minimal medium (MM) [5]
1.07 g/l NH_4Cl, 61.6 mg/l $MgSO_4 \cdot 7H_2O$, 10 g/l glucose, 10 ml/l 100 x M9 trace element solution, 1 ml/l vitamin mix (four vitamin mix or seven vitamin mix).

M9 trace elements (100 x stock) [5]
$Na_2EDTA \cdot 2H_2O$ (500 mg/l), $FeCl_2 \cdot 4H_2O$ (143 mg/l), $ZnCl_2$ (4.7 mg/l), $MnCl_2 \cdot 4H_2O$ (3 mg/l), H_3BO_3 (30 mg/l), $CoCl_2 \cdot 6H_2O$ (20 mg/l), $CuCl_2 \cdot 2H_2O$ (1 mg/l), $NiCl_2 \cdot 6H_2O$ (2 mg/l), $Na_2MoO_4 \cdot 2H_2O$ (3 mg/l), $CaCl_2 \cdot 2H_2O$ (100 mg/l).

Seven vitamin mix (1000 x stock) [6]
50 µg/l cyanocobalamin, 40 µg/l 4-aminobenzoic acid, 10 µg/l biotin, 100 µg/l nicotinic acid, 50 µg/l calcium pantothenate, 150 µg/l pyridoxamine dichloride, 100 µg/l thiamine dichloride.

Four Vitamin mix (1000 x stock)

100 µg/l thiamine dichloride, 50 µg/l cyanocobalamin, 100 µg/l nicotinic acid, 50 µg/l calcium pantothenate.

Pollen agar

20 g/l pollen (Bergland-Pharma GmbH & Co, Heimertingen, Germany), 1 g/l KH_2PO_4, 15g/l agar, adjusted to pH 5.5 with 2 N HCl.

Gas chromatography-mass spectrometry (GC-MS)

GC-MS was carried out using a Thermo Scientific Trace GC Ultra hyphenated to a Thermo Scientific ISQ mass spectrometer.

GC-MS conditions: GLC column: Macherey Nagel Optima 5MS 30 m x 0.25 mm, 0.25 µm, carrier gas: H_2, inlet temperature: 280 °C, injection volume: 0.1-1 µl.

MS conditions: Electron impact ionization (EI, 70 eV), ion source temperature 220 °C, MS transfer line temperature: 290 °C.

GC-MS program for the analysis of volatile organic compounds collected by SPME

Initial column temperature: 50 °C for 3 min; 6 °C/min to 200 °C, 20 °C/min to 260 °C, 260 °C for 1 min. Carrier gas flow rate 0.8 ml/min, split ratio: splitless, solvent delay 5.5 min. Mass range 41-300 Da.

GC-MS program for ethyl acetate extracts of spent *Rosenbergiella* medium

Initial column temperature: 50 °C; 20 °C/min to 80 °C; 10 °C/min to 280 °C, 280 °C for 1 min. Carrier gas flow rate: 0.7 ml/min, split ratio: 1:12, solvent delay 5 min. Mass range 41-450 Da.

Analysis of fatty acid methyl esters from *Rosenbergiella* strains by GC-MS

Lauric acid methyl ester: Rt = 9.00 min. EI-MS (m/z, (%)): 43 (28), 55 (27), 74 (100), 87 (55), 143 (12), 171 (10), 183 (9), 214 (6).

Myristic acid methyl ester: Rt = 11.23 min. EI-MS (m/z, (%)): 43 (26), 55 (23), 74 (100), 87 (59), 143 (16), 199 (15), 211 (8), 242 (12).

Hexadecenoic acid methyl ester: Rt = 13.05 min. EI-MS (m/z, (%)): 43 (45), 55 (100), 69 (65), 96 (53), 152 (14), 194 (7), 236 (25), 268 (8).

Palmitic acid methyl ester: Rt = 13.28 min. EI-MS (m/z, (%)): 43 (28), 55 (22), 74 (100), 87 (65), 143 (14), 227 (11), 270 (15).

Saturated C17 cyclopropyl fatty acid methyl ester: Rt = 14.08 min. EI-MS (m/z, (%)): 55 (100), 69 (67), 74 (54), 83 (48), 96 (43), 111 (23), 166 (10), 208 (12), 250 (18), 282 (3).

Octadecenoic acid methyl ester: Rt = 14.96 min. EI-MS (m/z, (%)): 55 (100), 69 (67), 96 (44), 111 (22), 180 (10), 222 (14), 264 (25), 296 (5).

Stearic acid methyl ester: Rt = 15.14 min. EI-MS (m/z, (%)): 43 (39), 55 (31), 74 (100), 87 (63), 143 (16), 255 (11), 298 (20).

Table S1: Fatty acid methyl esters from lipid hydrolysates of *Rosenbergiella* isolates and *R. epipactidis* 2.1A. Numbers represent peak area percentage of the total integrated area. Only compounds with peak areas > 5 % are listed. Compounds were identified by comparisons to authenticated standards and/or NIST library comparisons of the fragmentation patterns.

**R_T (min)**	**Identity**	***R. epipactidis* 2.1A**	***Rosenbergiella* sp. D8K**	***Rosenbergiella* sp. D15G**	***R. meliponini* D21B**
9.00	Lauric acid	Trace	Trace	Trace	Trace
11.23	Myristic acid	6	13	11	6
13.05	Palmitoleic acid	Trace	Trace	Trace	Trace
13.28	Palmitic acid	64	68	64	64
14.08	$C_{17:0\ cyclo}$	12	5	20	12
14.96	Octadecenoic acid	9	Trace	Trace	8
15.14	Stearic acid	6	Trace	Trace	6

Fatty acid trimethylsilyl esters from lipid hydrolysates of *Rosenbergiella* isolates and *R. epipactidis* 2.1A

All *Rosenbergiella* strains examined here exhibited the following five main peaks.

Lauric acid trimethylsilyl ester: Rt = 10.52 min. EI-MS (m/z, (%)): 73 (90), 75 (71), 117 (100), 129 (36), 132 (46), 145 (18), 257 (89), 272 (6).

Myristic acid trimethylsilyl ester: Rt = 12.58 min. EI-MS (m/z, (%)): 73 (83), 75 (66), 117 (100), 129 (36), 132 (47), 145 (21), 285 (83), 300 (8).

3-(Trimethylsiloxy)myristic acid trimethylsilyl lester: Rt = 14.40 min. EI-MS (m/z, (%)): 73 (100), 75 (43), 147 (58), 233 (68), 257 (29), 373 (20).

Palmitic acid trimethylsilyl ester: Rt = 14.47 min. EI-MS (m/z, (%)): 73 (82), 75 (59), 117 (100), 129 (41), 132 (54), 145 (24), 313 (65), 328 (9).

C17 Cyclopropyl fatty acid trimethylsilyl ester: Rt = 15.22 min. EI-MS (m/z, (%)): 55 (78), 73 (87), 75 (100), 117 (79), 129 (51), 145 (29), 325 (31).

Table S2: Fatty acid TMS esters from lipid hydrolysates of *Rosenbergiella* isolates and *R. epipactidis* 2.1A. Numbers represent peak area percentage total integrated area. Only compounds with peak areas > 5% are listed. Compounds were identified by comparisons to known standards and/or NIST library comparison of fragmentation patterns.

R_T (min)	Identity	*R. epipactidis* 2.1A	*Rosenbergiella* sp. D08K	*Rosenbergiella* sp. D15G	*R. meliponini* D21B
10.52	Lauric acid	12	26	14	14
12.58	Myristic acid	12	21	17	18
14.40	$C_{14:0\text{-}3\text{-}OH}$	41	28	29	35
14.47	Palmitic acid	25	18	28	24
15.21	$C_{17:0\ cyclo}$	10	8	12	9

Compounds identified by GC-MS in ethyl acetate extracts of spent medium of *R. meliponini* D21B

2-Phenylethanol trimethylsilyl ether: Rt = 5.46 min. EI-MS (m/z, (%)): 73 (100), 75 (37), 91 (31), 103 (88), 161 (12), 179 (72), 180 (12).

2-Phenylacetic acid trimethylsilyl ester: Rt = 6.31 min. EI-MS (m/z, (%)):65 (7), 73 (100), 75 (34), 91 (17), 164 (22), 193 (12), 194 (2).

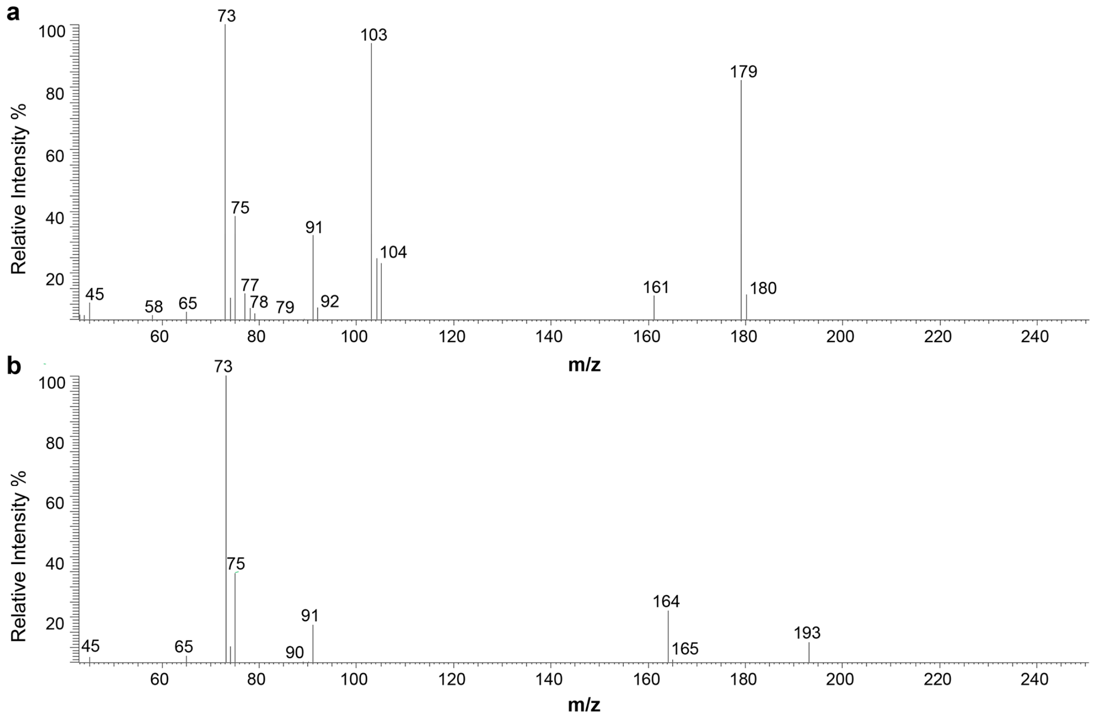

Figure S1: EI-MS spectra of the major compounds in the spent medium of *R. meliponini* 2.1A, (a) 2-phenylethanol, (b) 2-phenylacetic acid

GC-MS analysis of volatile organic compounds released by the *Rosenbergiella* isolates and *R. epipactidis* 2.1A

2-Phenylethanol, Rt = 9.60 min. EI-MS (m/z, (%)): 65 (17), 77 (5), 78 (6), 91 (100), 92 (61), 104 (7), 122 (31).

2-Phenylethanol acetate, Rt = 13.02 min. EI-MS (m/z, (%)): 43 (37), 65 (5), 78 (8), 91 (14), 104 (100), 105 (11).

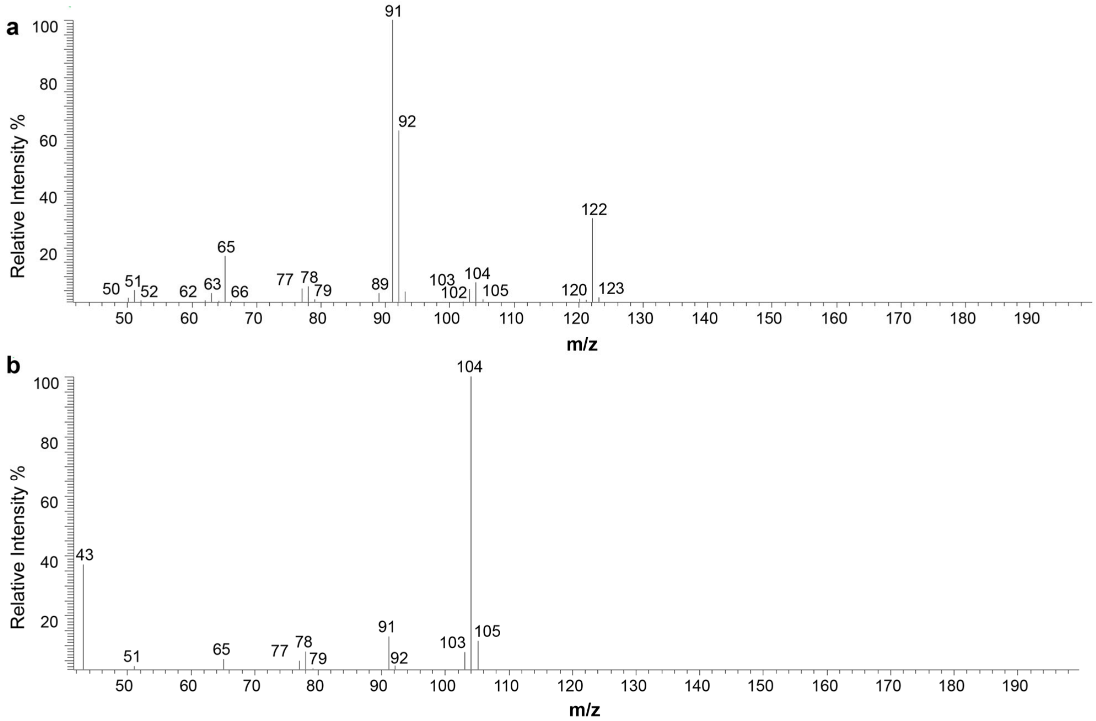

Figure S2: EI-MS spectra of (a) 2-phenylethanol and (b) 2-phenylethyl acetate

Quantification of 2-phenylethanol and 2-phenylacetic acid

A standard curve of 2-phenylethanol and 2-phenylacetic acid (each at 10 mg/ml in acetone) was prepared, using benzoic acid (0.86 mg/ml in acetone) as an internal standard. Varying quantities (1-10 µl) of 2-phenylethanol/2-phenylacetic acid solution were combined with benzoic acid standard solution (50 µl) and the solvent was evaporated at 60 °C on a heat block. MSTFA (10 µl) was added, the vial was capped, and the mixture heated at 60 °C for 1 h. Diethyl ether (ca. 100 µl) was added and the mixture was analysed by GC-MS.

R. meliponini D21B and *R. epipactidis* 2.1A were each grown for 6 d in RYS broth (100 ml, 28 °C, 150 rpm). Cells were pelleted by centrifugation (RCF 4162, 20 min).

The supernatant was acidified with 2N HCl (pH 1-2) and extracted with diethyl ether (3 x 20 ml). The extracts were dried and the solvent was reduced to approx. 20 ml by rotary evaporation and subsequently made up accurately to 40 ml. Aliquots of these extracts were combined with the aforementioned benzoic acid standard solution (50 µl), derivatized with MSTFA as described above, and analyzed by GC-MS.

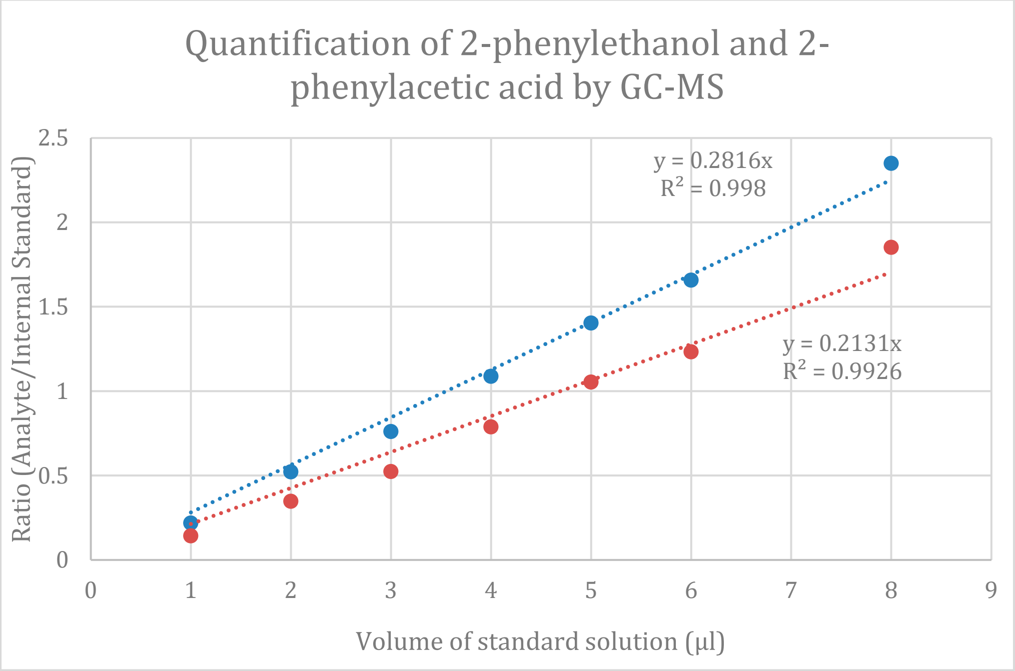

Figure S3: Standard curve of 2-phenylethanol (blue) and 2-phenylacetic acid (red) by GC-MS (internal standard: benzoic acid)

Table S3: Quantification of 2-phenylethanol and phenylacetic acid in spent medium extracts of *R. epipactidis* 2.1A and *R. meliponini* D21B by GC-MS. Average TIC (total ion count) peak area ratios of the analytes are divided by the internal standard (benzoic acid). The averages and standard deviations of four replicates are shown.

Extract	Volume (µl)	2-Phenylethanol		2-Phenylacetic acid	
		Average TIC ratio (SD)	Concentration in spent medium (SD)	Average TIC ratio (SD)	Concentration in spent medium (SD)
***R. epipactidis* 2.1A**	80	0	0	0.8198 (0.0819)	0.192 g/l (0.019)
***R. meliponini* D21B**	100	1.2482 (0.0622)	0.177 g/l (0.009)	0.1325 (0.0121)	(Below range of standard curve)
	150	2.3215 (0.1577)	(Above range of standard curve)	0.2312 (0.0082)	0.029 g/l (0.001)

Phylogenetic analysis of 16*S* rDNA and house-keeping genes of *Rosenbergiella* isolates from *T. carbonaria*

Detailed neighbour joining phylogenetic trees of *Rosenbergiella* strains

Just before the submission of our manuscript, two new *Rosenbergiella* species, *R. metrosideri* strain JB07) and *R. gaditana* strain S61, were reported [7]. To date, the housekeeping genes 16S, *rpo*B, *atp*D and gyrB have been published, but the full or draft genomes are not yet publicly available. The similarity in the sequences of some of the housekeeping genes between *R. meliponini* D21B and *R. metrosideri* JB07 in particular warrants further investigation (Figure A4-A7, Table S5). Nevertheless, their substantial biochemical differences (see Table S8) justify the classification of these two organisms as different species despite the similarities in their housekeeping genes.

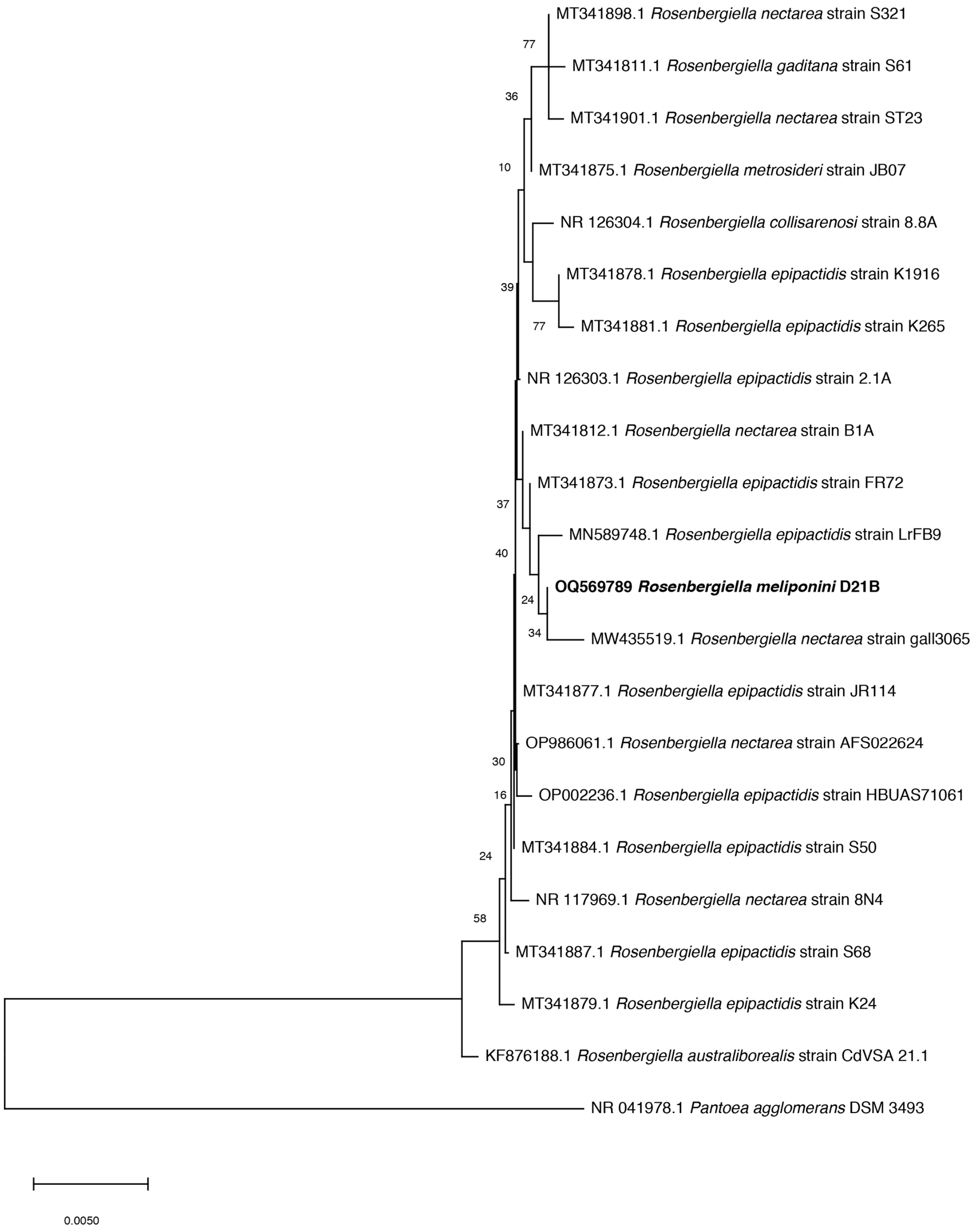

Figure S4: Neighbor-joining phylogenetic tree based on 16*S* rRNA gene sequences (Clustal W alignment), including *R. metrosideri* strain JB07. Bootstrap values calculated from 1000 replicates are indicated at branching nodes. Scale bar indicates 0.005 substitutions per nucleotide position.

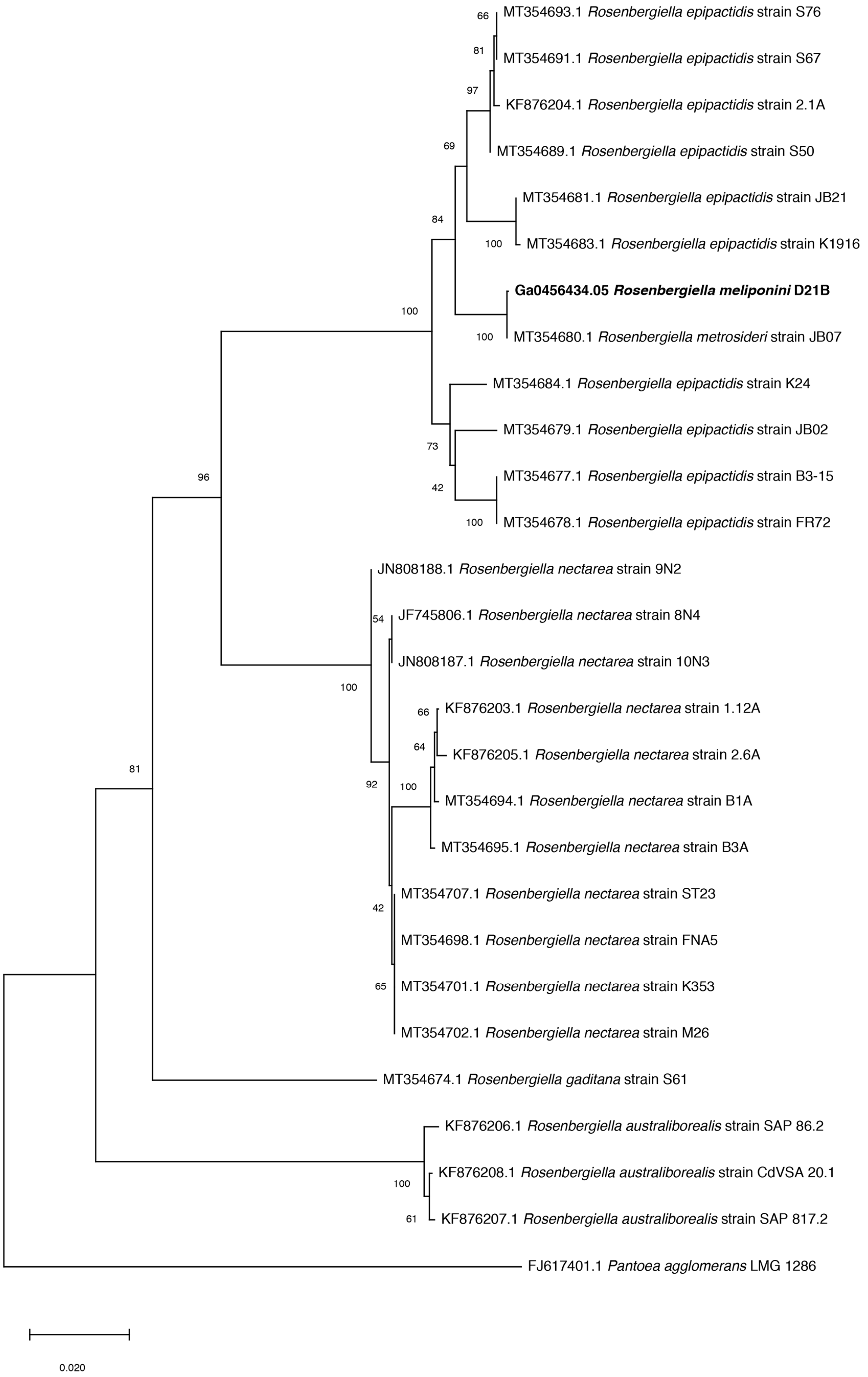

Figure S5: Neighbor-joining phylogenetic tree based on gyrB sequences (Clustal W alignment), including the recently reported *R. metrosideri* strain JB07. Bootstrap values calculated from 1000 replicates are indicated at branching nodes. Scale bar indicates 0.02 substitutions per nucleotide position.

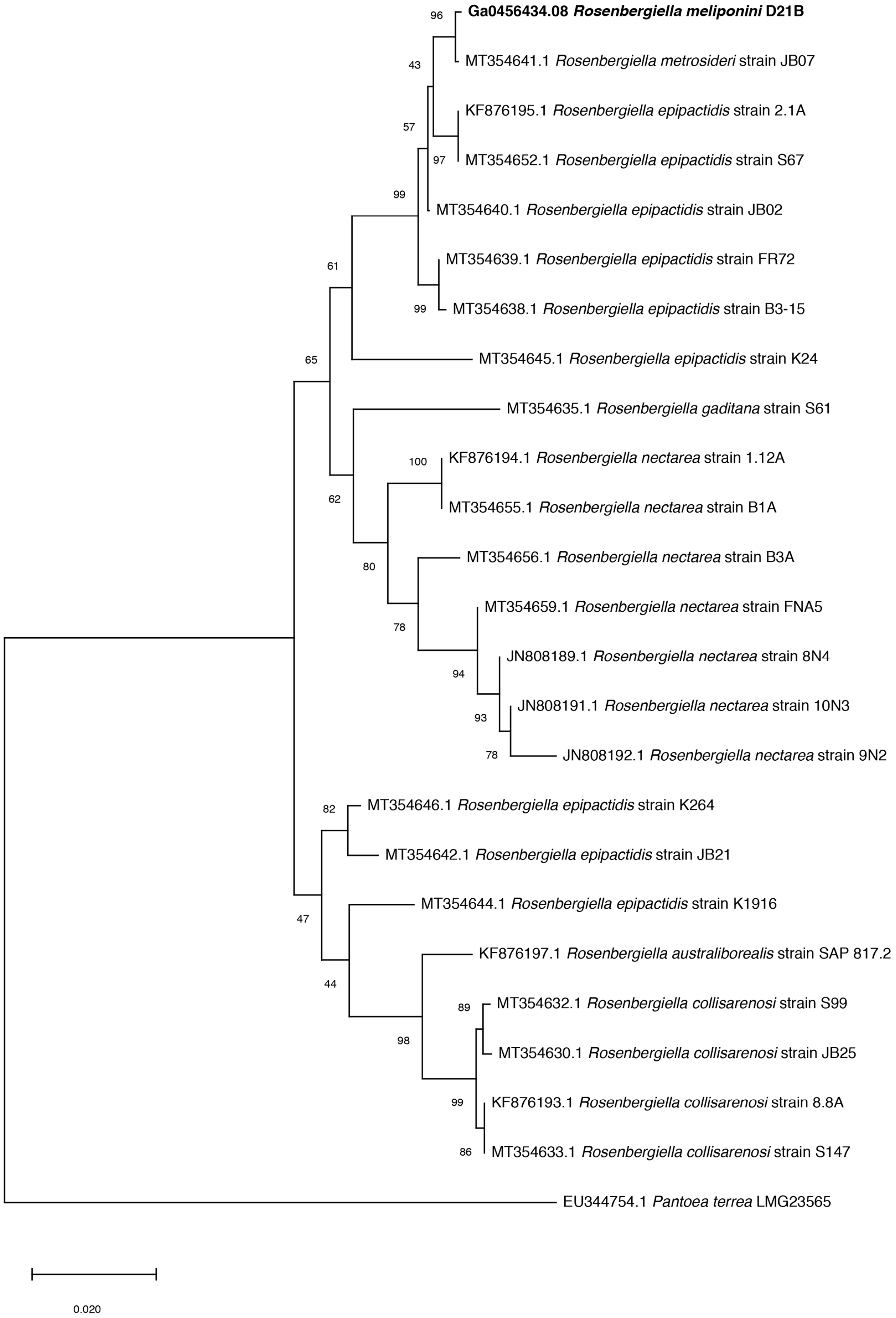

Figure S6: Neighbor-joining phylogenetic tree based on atpD gene sequences (Clustal W alignment), including *R. metrosideri* strain JB07. Bootstrap values calculated from 1000 replicates are indicated at branching nodes. Scale bar indicates 0.02 substitutions per nucleotide position.

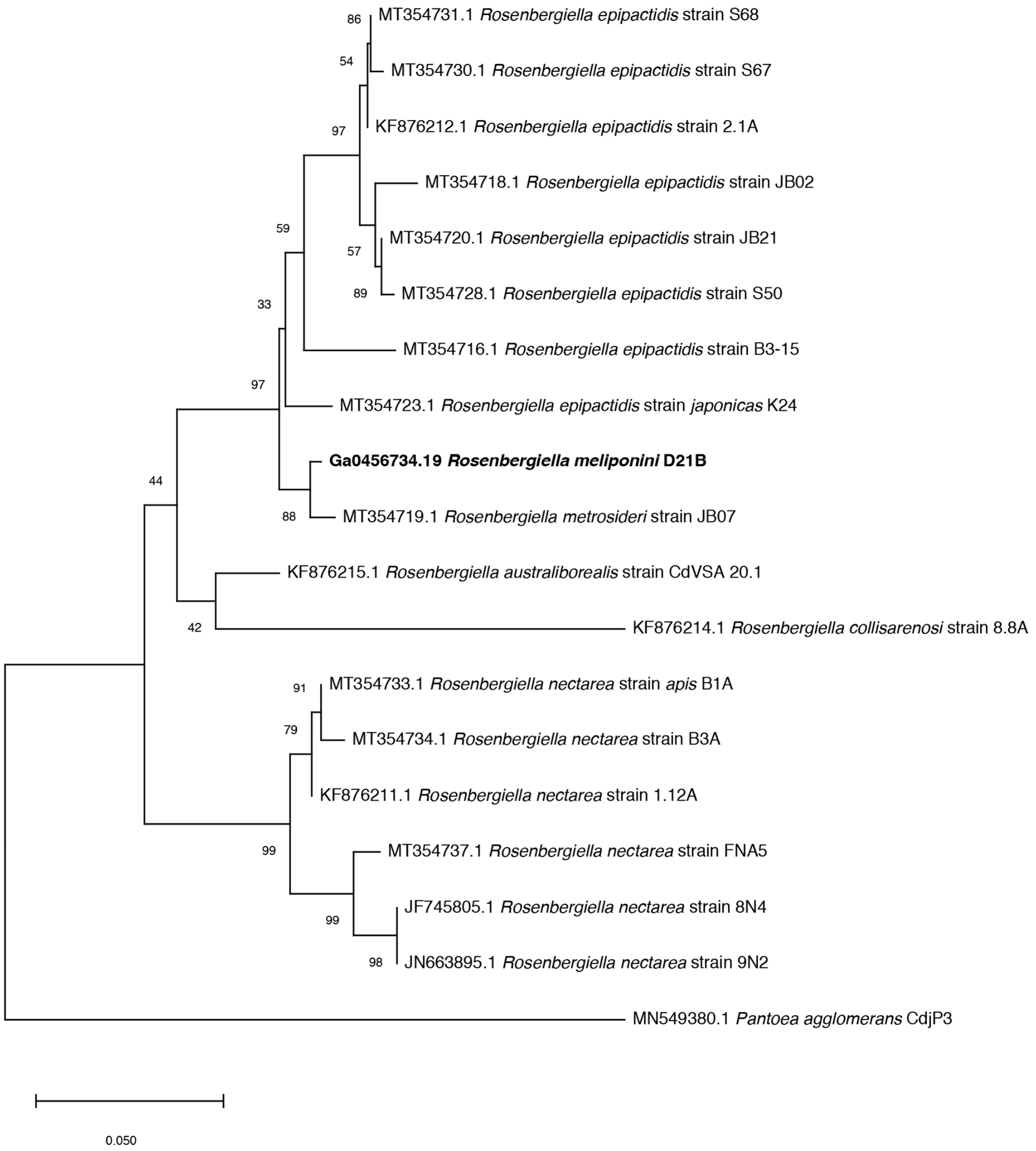

Figure S7: Neighbor-joining phylogenetic tree based on *rpoB* gene sequences (Clustal W alignment), including *R. metrosideri* strain JB07. Bootstrap values calculated from 1000 replicates are indicated at branching nodes. Scale bar indicates 0.05 substitutions per nucleotide position.

ANI scores

Table S4: ANI scores [8] of *R. meliponini* D21B against sequenced *Rosenbergiella* strains, SD standard deviation.

ANI score of	*R. nectarea* 8N4	*R. epipactidis* 2.1A	*R. australiborealis* CdVSA 20.1	*R. collisarenosi* 8.8A
R. meliponini D21B versus	85.82 (SD, 4.21 %)	94.84 (SD, 2.82 %)	94.84 (SD, 2.82 %)	80.34 (SD, 5.77 %)

Table S5: Comparison of the phylogenetic analysis of *Rosenbergiella* isolates from *T. carbonaria* with other *Rosenbergiella* strains.

	***Rosenbergiella* strain**	*R. meliponini* D21B	*R. epipactidis* 2.1A	*R. nectarea* 8N4	*R. australiborealis* CdVSA20.1	*R. collisarenosi* 8.8A	*R. metrosideri* JB07
**16*S* rDNA**	*R. meliponini* D21B		99.63 %	99.46 %	99.66 %	99.80 %	99.85 %
**16*S* rDNA**	*Rosenbergiella* sp. D08K	99.78 %	99.78 %	99.40 %	99.27 %	99.40 %	99.85 %
**16*S* rDNA**	*Rosenbergiella* sp. D15G	99.77 %	99.85 %	99.47 %	99.54 %	99.69 %	99.85 %
**gyrB**	*R. meliponini* D21B		97.26 %	87.43 %	82.21 %	81.19 %	100 %
atpD	*R. meliponini* D21B		99.19 %	96.08 %	94.87 %	94.20 %	99.87 %
ropB	*R. meliponini* D21B		97.43 %	90.30 %	93.86 %	86.93 %	99.07 %

Characteristics of the draft genome of *R. meliponini* D21B

Table S6: Distribution of clusters of orthologous genes in the draft genome of *R. meliponini* D21B (COGs)

**Name**	**Number of genes**	**Percentage of all identified genes**
Amino acid transport and metabolism	302	10.17 %
Carbohydrate transport and metabolism	188	6.33 %
Cell cycle control, cell division, chromosome partitioning	43	1.45 %
Cell motility	113	3.81 %
Cell wall/membrane/envelope biogenesis	221	7.44 %
Coenzyme transport and metabolism	158	5.32 %
Cytoskeleton	1	0.03 %
Defence mechanisms	74	2.49 %
Energy production and conversion	139	4.68 %
Extracellular structures	26	0.88 %
Function unknown	155	5.22 %
General function prediction only	252	8.49 %
Inorganic ion transport and metabolism	196	6.60 %
Intracellular trafficking, secretion, and vesicular transport	84	2.83 %
Lipid transport and metabolism	87	2.93 %
Mobilome: prophages, transposons	24	0.81 %
Nucleotide transport and metabolism	83	2.80 %
Posttranslational modification, protein turnover, chaperones	120	4.04 %
RNA processing and modification	1	0.03 %
Replication, recombination and repair	117	3.94 %
Secondary metabolites biosynthesis, transport and catabolism	58	1.95 %
Signal transduction mechanisms	101	3.40 %
Transcription	191	6.43 %
Translation, ribosomal structure and biogenesis	235	7.92 %
Genes not assigned into an abovementioned COG category	529	17.50 %

Table S7: Minimum inhibitory concentrations of selected antibiotics against *Rosenbergiella* strains. *E. coli* Top10 serves as a reference antibiotic-sensitive *Enterobacterium*. Values are in µg/ml, (n = 3).

**Strain**	**Ampicillin**	**Kanamycin**	**Chloramphenicol**	**Novobiocin**
R. meliponini D21B	300	17	20	67
Rosenbergiella sp. D15G	300	17	20	67
Rosenbergiella sp. D08K	100	6	20	22
R. epipactidis 2.1A	300	6	20	67
E. coli Top10	33	6	2	200
Standard conc. [9-11]	100	50	20	200

Table S8: Morphological and biochemical characterization of *R. meliponini* D21B in comparison to the four reported *Rosenbergiella* type strains as well as the recently reported *R. metrosideri* JB07, including EnteroPluri biochemical results. Legend: + positive reaction, − negative reaction, w weak, n/a denotes data not available. Data for *R. meliponini* D21B, *Rosenbergiella* sp. D08K, *Rosenbergiella* sp. D15G and *R. epipactidis* 2.1A were derived experimentally. Data for *R. collisarenosi* 8.8A, *R. australiborealis* CdBSA20.1, *R. nectarea* 8N4 and *R. metrosideri* JB07 were obtained from the literature [7,12,13]. Substantial biochemical differences between *R. meliponini* D21B and the closest type strains according to housekeeping gene analysis, *R. epipactidis* 2.1A and *R. metrosideri* JB07, are shown in bold.

**Characteristic**	***R. meliponini* D21B**	***Rosenbergiella* sp. D08K**	***Rosenbergiella* sp. D15G**	***R. epipactidis* 2.1A**	***R. collisarenosi* 8.8A**	***R. australiborealis* CdBSA20.1**	***R. nectarea* 8N4**	***R. metrosideri* JB07**
Colony colour (on LB, J, RYS agar)	Yellow/orange	Yellow/orange	Yellow/orange	Yellow/orange	Yellow/orange	Yellow/orange	Yellow/orange	Pale yellow
Gram stain	−	−	−	−	−	−	−	−
Cell morphology	Rods	Rods	Rods	Rods	Rods	Rods	Rods	Rods
Motility	Motile	Motile	Motile	Motile	Motile	Motile	Motile	Motile
Oxidase activity	−	−	−	−	−	−	−	−
Catalase activity	+	+	+	+	+	+	+	+
Anaerobic growth	Facultative	Facultative	Facultative	Facultative	Facultative	Facultative	Facultative	Facultative
Sucrose tolerance	0-50 %	0-50 %	0-50 %	0-50 %	0-50 %	0-50 %	0-50 %	0-50 %
Growth at 4 °C	−	−	−	−	−	Not reported	−	w
Growth at 28 °C	+	+	+	+	n/a	n/a	n/a	+

**Characteristic**	***R. meliponini* D21B**	***Rosenbergiella* sp. D08K**	***Rosenbergiella* sp. D15G**	***R. epipactidis* 2.1A**	***R. collisarenosi* 8.8A**	***R. australiborealis* CdBSA20.1**	***R. nectarea* 8N4**	***R. metrosideri* JB07**
**Growth at 37 °C**	**Poor**	**Poor**	**Poor**	**−**	**−**	**+**	**+**	**+**
Glucose/gas	+/−	+/−	+/−	+/−	+	+	+	+, n/a
**Lysine decarboxylase**	**−**	**−**	**−**	**−**	**n/a**	**n/a**	**−**	**+**
Ornithine decarboxylase	−	−	−	−	n/a	n/a	−	−
Production of H_2S	−	−	−	−	−	−	−	−
Indole	−	−	−	−	−	−	−	−
Adonitol	−	−	−	−	−	−	−	−
Lactose	−	−	−	−	−	−	−	−
**Arabinose**	**+**	**+**	**+**	**+**	**+**	**+**	**+**	**−**
Sorbitol	−	−	−	−	−	−	−	−
**Acetoin**	**+**	**+**	**+**	**+**	**+**	**+**	**+**	**−**
Dulcitol	−	−	−	−	−	−	−	n/a
Phenylalanine	−	−	−	−	n/a	n/a	n/a	n/a
**Urease**	**W**	**+**	**w**	**−**	**n/a**	**n/a**	−	−
**Citrate**	**+**	**+**	**+**	**−**	**−**	**−**	**−**	**−**

Secondary metabolite biosynthetic gene clusters from *R. meliponini* D21B

AntiSMASH analysis [14,15] revealed five secondary metabolite gene clusters in *R. meliponini* D21B (see Figure A8 and Table S9).

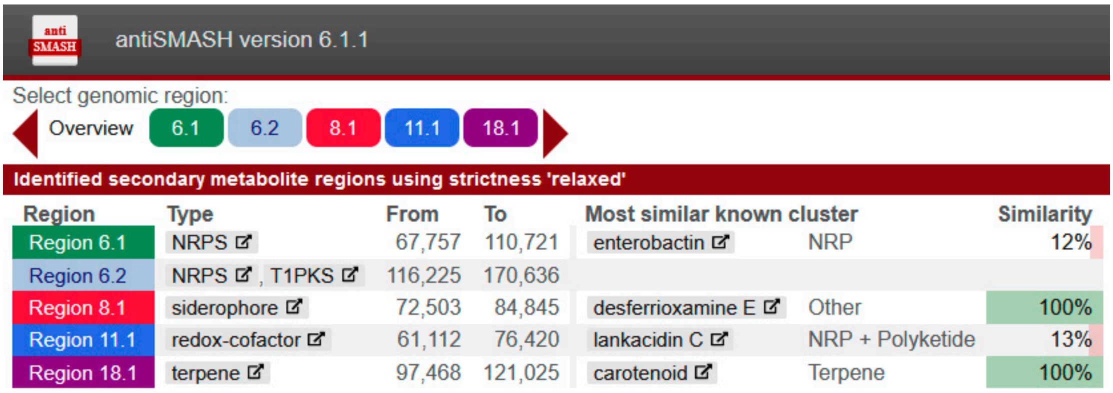
antiSMASH version 6.1.1

Select genomic region:

Overview 6.1 6.2 8.1 11.1 18.1

Identified secondary metabolite regions using strictness 'relaxed'

Region	Type	From	To	Most similar known cluster		Similarity
Region 6.1	NRPS	67,757	110,721	enterobactin	NRP	12%
Region 6.2	NRPS , T1PKS	116,225	170,636			
Region 8.1	siderophore	72,503	84,845	desferrioxamine E	Other	100%
Region 11.1	redox-cofactor	61,112	76,420	lankacidin C	NRP + Polyketide	13%
Region 18.1	terpene	97,468	121,025	carotenoid	Terpene	100%

Figure S8: Summary of secondary metabolite biosynthetic gene clusters of *R. meliponini* D21B detected by antiSMASH [14,15].

Cluster 8.1 is predicted to encode for the production of the siderophore desferrioxamine (Figure A2) with 100 % similarity [16].

The biosynthetic gene cluster 6.1 is highly conserved across the *Rosenbergiella* genus, and shares low similarity to the biosynthetic gene clusters for the production of enterobactin like siderophores (Figure A4). It contains 10 out of 12 genes that are responsible for enterobactin synthesis in *E. coli* K12 [17]. However, both *R. meliponini* D21B and *R. nectarea* 8N4 lack genes similar to the lipopolysaccharide (LPS) O-antigen chain length determinant protein (FepE), and a gene that encodes an enterobactin exporter of the ENTS family (EntS).

Cluster 6.2 is predicted to code for a polyketide synthase non-ribosomal peptide synthetase biosynthetic gene cluster that is unique to *Rosenbergiella* D21B and may produce an unknown secondary metabolite.

Cluster 8.1 encodes genes necessary for the production of desferrioxamine, and this cluster is conserved across all *Rosenbergiella* genomes currently available.

Cluster 11.1 encodes for a putative redox cofactor of unknown function that appears to be conserved among the *Rosenbergiella* strains.

Cluster 18.1 is predicted to code for the production of carotenes with 100 % similarity (see chapter vitamins).

Table S9: Secondary metabolite producing gene cluster positions predicted by AntiSMASH [14,15]

**Gene clusters predicted in *R. meliponini* D21B1**	***R. epipactidis* JB21**	***R. epipactidis* S68**	***R. epipactidis* JB02**	***R. epipactidis* GCF02260**	***R. nectarea* GCF9001**
6.1 (enterobactin)	29.2	30.1	45.1	31.1	19.1
6.2 (NRPS/T1PKS)	-	-	-	-	-
8.1 (desferrioxamine)	32.1	2.1	4.1	37.1	9.1
11.1 (redox cofactor of unknown function)	4.1	29.1	10.1	14.1	18.1
18.1 (terpene /carotenoid)	6.1	32.1	16.1	6.1	16.1

It should be noted that for all gene sequence comparisons except for the four housekeeping genes that were examined for the purpose of establishing the phylogenetic placement of the novel bacteria species, the putative peptide sequence identity was used for comparative purposes. Due to the degenerate nature of the genetic code, percentage peptide sequence identity was thought to provide a more meaningful comparison of gene conservation between species of bacteria.

Table S10: Conservation of desferrioxamine producing genes in *R. meliponini* D21B and the four reported *Rosenbergiella* species. Genes which are different in *R. meliponini* D21B to the other sequenced *Rosenbergiella* strains are highlighted in bold. The percentage of sequence identities is calculated for the putative protein sequence. Comparisons below have been made against characterized *dfo* genes from *Erwinia amylovora* (strain CFBP1430) [18,19]. Y indicates the presence of the gene.

**Characterized gene**	**Locus tags**	**% Sequence identity**	***R. meliponini* D21B**	***R. nectarea***	***R. epipactidis***	***R. australiborealis***	***R. collisarenosi***
dfoJ, pyridoxal-dependent decarboxylase	Ga0456434_08_ 74680_76209	65%	Y	Y	Y	Y	Y
dfoA, FAD-dependent amine monooxygense	Ga0456434_08_ 76209_77510	64%	Y	Y	Y	Y	Y
dfoC (*desC, desD*), desferrioxamine synthetase	Ga0456434_08_ 77503_79845	58%	Y	Y	Y	Y	Y
FoxR, siderophore uptake transmembrane transporter	Ga0456434_08_ 81185_83302	72%	Y	Y	Y	Y	Y

In addition to the prediction by antiSMASH the genome of *R. meliponini* D21B was screened for the presence of pyocin and colicin genes because these genes have been identified in other *Rosenbergiella* strains. Only the colicin V production gene cvpA was found in the *R. meliponini* D21B draft genome.

Table S11: Conservation of possible pyocin and colicin encoding genes in *Rosenbergiella* strains. Y indicates the presence of the gene, N indicates its absence

	**Characterized gene**	**Locus tags**	**% Sequence identity**	***R. meliponini* D21B**	***R. nectarea***	***R. epipactidis***	***R. australiborealis***	***R. collisarenosi***
Pyocin	S-type pyocin domain-containing protein coding gene (X5) (from *R, nectarea* 8N4)			N	Y	N	N	N
Colicin	Colicin uptake gene tolR			N	Y	Y	Y	Y
	colicin V production gene cvpA (of *Rosenbergiella epipactidis* 2.1A) (WP_048912330.1)	Ga0456434_01_235077_235562	n/a	Y	N	Y	Y	Y
	colicin immunity	-		N	N	N	Y	Y
	colicin-E6	-		N	N	N	Y	N
	tellurite/colicin resistance gene	-		N	N	N	N	Y

Table S12: Comparison of carbohydrate utilization by *Rosenbergiella* strains (additional tests to those in the EnteroPluri tubes). + Indicates the production of acid.

**Carbohydrate**	***R. meliponini* D21B**	***Rosenbergiella* sp. D08K**	***Rosenbergiella* sp. D15G**	***R. epipactidis* 2.1A**	***R. collisarenosi* 8.8A**	***R. australiborealis* CdVSA20.1**	***R. nectarea* 8N4**
D-Fructose	+	+	+	+	+	+	+
D-Galactose	+	+	+	+	+	+	+
D-Mannose	+	+	+	+	+	−	+
D-Ribose	+	+	+	+	+	−	+
D-Lyxose	−	−	−	−	−	−	−
D-Mannitol	−	−	−	−	−	−	−
D-Maltose	−	−	−	−	−	−	−
Sucrose	+	+	+	+	+	+	+
Glycerol	+	+	+	+	+	+	+
Trehalose	−	−	−	−	−	−	−
Arabinogalactan	−	−	−	−	n/a	n/a	n/a

Amino acid biosynthesis by *Rosenbergiella* strains

All *Rosenbergiella* isolates from *T. carbonaria* as well as *R. epipactidis* A2.1 could grow in minimal media without addition of amino acids, proving that the investigated *Rosenbergiella*can produce all 20 proteinogenic amino acids.

Production of vitamins by *Rosenbergiella* strains

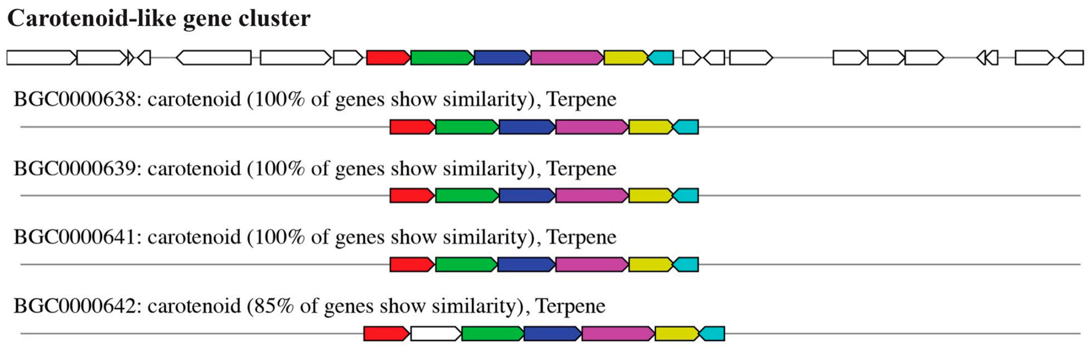

Figure S9: *R. meliponini* D21B comprises a carotenoid biosynthetic gene cluster with 100 % similarity to known carotenoid biosynthetic gene clusters[20].

Analysis by antiSMASH [14,15] revealed that *R. meliponini* D21B draft genome contains the full set of genes required for carotenoid biosynthesis (Figure A9 and Table S13). For comparative purposes, the genome assemblies of other four *Rosenbergiella* type strains (*R. epipactidis* 2.1A, *R. nectarea* 8N4, *R. australiborealis* CdVSA20.1 and *R. collisarenosi* 8.8A) were examined. The carotenoid-like gene cluster of *R. meliponini* D21B that was detected at the end of scaffold 18 (Ga0456434_18_104611_111500) was present in all four *Rosenbergiella* type strains. The first gene was identified to code for an YtfJ family protein, and exhibited 56 % peptide sequence identity to the corresponding gene in *Pantoea ananatis* PA13[20], a transcriptional regulator. The following six genes showed 57 %, 52 %, 59 %, 82 %, 63 %, and 66 % sequence identity to carotenoid biosynthetic genes *ctrE*, *ctrX*, *ctrY*, *ctrl*, *ctrB*, and *ctrZ* in *Pantoea ananatis,* respectively [20] (Table S13). A gene downstream of *ctrEXYIBZ* cluster (Ga0456434_07_108871_109395) exhibited 80 % sequence conservation with *idi* gene encoding the isopentenyl diphosphate isomerase in *Pantoea* species, an enzyme that produces the common precursor farnesyl diphosphate (FPP) [21].

Table S**13:** Carotenoid biosynthetic genes in *R. meliponini* D21B and the four reported *Rosenbergiella* species with highest sequence identity to the putative protein sequence to *P. ananatis* PA13 carotenoid biosynthetic genes [20]. Legend: Y indicates a highly conserved gene.

**Gene**	**Locus tag**	**% Sequence identity (putative peptide sequence)**	***R. meliponini* D21B**	***R. nectarea***	***R. epipactidis***	***R. australiborealis***	***R. collisarenosi***
transcriptional regulator	Ga0456434_18_104611_105198	56 %	Y	Y	Y	Y	Y
ctrE	Ga0456434_18_105277_106185	57 %	Y	Y	Y	Y	Y
ctrX	Ga0456434_18_106195_107475	52 %	Y	Y	Y	Y	Y
ctrY	Ga0456434_18_107468_108616	59 %	Y	Y	Y	Y	Y
ctrl	Ga0456434_18_108625_110106	82 %	Y	Y	Y	Y	Y
ctrB	Ga0456434_18_110099_111025	63 %	Y	Y	Y	Y	Y
ctrZ	Ga0456434_18_110970_111500	66 %	Y	Y	Y	Y	Y
idi, isopentenyl diphosphate isomerase	Ga0456434_07_108871_109395	80 %	Y	Y	Y	Y	Y

Vitamin B_1 (thiamine)

While some bacteria synthesize thiamine [22,23], some enteric bacteria salvage thiazole (hydroxyethylthiazole; HET) and pyrimidine (hydroxymethylpyrimidine; HMP) from the culture medium using enzymes encoded by *thiM* and *thiD* respectively and produce thiamine monophosphate [24]. Genes that encode for vitamin B_1 synthesizing enzymes (ThiO, ThiJ and TenI) were not found in *R. nectarea*, *R. epipactidis*, *R. australiborealis* and *R. collisarenosi*. However, putative thiamine biosynthetic genes, albeit with low peptide sequence similarity (26%, 41% and 28%, respectively) to ThiO, ThiJ and TenI, were found in the *R. meliponini* draft genome (Table S15). Moreover, *R. meliponini* D21B grew in minimal medium without thiamine, whereas *R. epipactidis* 2.1A grew poorly without thiamine supplementation (see Table S14).

Table S14: Growth of *Rosenbergiella* strains (determined by OD_{600} measurement) in minimal medium [6] with and without supplementation of vitamin B_1, vitamin B_3, vitamin B_5 and vitamin B_{12}.

**Vitamin(s) supplemented**	***R. meliponini* D21B**	***Rosenbergiella* sp. D08K**	***Rosenbergiella* sp. D15G**	***Rosenbergiella epipactidis* 2.1A**
None	0.02	0.05	0.03	0.01
B_3	0.65	0.56	0.61	0.08
B_1, B_3	0.76	0.60	0.68	1.06
B_1, B_3, B_5, B_{12}	1.02	0.99	0.93	1.33

Table S**15:** Vitamin B_1 biosynthetic genes in *R. meliponini* D21B and the four reported *Rosenbergiella* species with % peptide sequence identity to *E. coli* K12 proteins [22,23]. Legend: Y indicates the presence of a highly conserved gene, and N indicates the absence of a similar gene.

**Gene**	**Locus Tag**	**% putative peptide sequence identity**	***R. meliponini* D21B**	***R. nectarea* 8N4**	***R. epipactidis* 2.1A**	***R. australiborealis* CdVSA 20.1**	***R. collisarenosi* 8.8A**
Dxs, 1-deoxy-D-xylulose-5-phosphate synthase	Ga0456434_02_36603_38468	76 %	Y	Y	Y	Y	Y
PLP cysteine, pyridoxal-5'-phosphate dependant cysteine			N	N	N	N	N
ThiI, [ThiS-adenylate] sulfur transferase	Ga0456434_02_39846_41294	79%	Y	Y	Y	Y	Y
ThiS, sulfur carrier protein			N	Y	Y	N	Y
IscS, cysteine desulfurase	Ga0456434_01_46953_48167	87%	Y	Y	Y	Y	Y
ThiF, sulfur carrier protein ThiS adenylyltransferase	Ga0456434_07_97357_98115	40%	Y	Y	Y	Y	Y
ThiO, glycine oxidase (*Bacillus subtilis* - strain 168)	Ga0456434_08_92883_93827	26%	Y	N	N	N	N
ThiH, 2-iminoacetate synthase			N	N	N	N	N
ThiG, thiazole synthase	Ga0456434_08_94012_94770	41	Y	N	N	N	N
TenI, transcriptional regulator (*Bacillus subtilis* - strain 168)	Ga0456434_19_13551_14174	28	Y	N	N	N	N
ThiC, phosphomethylpyrimidine synthase	Ga0456434_19_14176_16098	64	Y	Y	Y	N	Y
ThiD, hydroxymethyl pyrimidine (phosphate) kinase	Ga0456434_10_387234_388052	41	Y	Y	Y	N	Y
ThiE, thiamine phosphate synthase	Ga0456434_19_13551_14174	31	Y	Y	Y	N	Y
ThiL, thiamine phosphate kinase	Ga0456434_02_35581_36576	36	Y	Y	Y	Y	Y

Vitamin B_2 (Riboflavin)

The riboflavin biosynthesis pathway has been studied in *E. coli* K12 [25]. All *Rosenbergiella* genomes contain genes with high similarity to *ribA*, *ribD*, and *ribE*, but *ribH* was not consistently identified across all *Rosenbergiella* genomes (Table S16). Both *R. meliponini* D21B and *R. epipactidis* 2.1A could grow in minimal medium without riboflavin supplementation, suggesting that both *Rosenbergiella* strains can produce riboflavin (Table S14).

Table S**16:** Vitamin B_2 biosynthetic genes in *R. meliponini* D21B and the four reported *Rosenbergiella* species with % of putative protein sequence identity to the *E. coli* K12 vitamin B_2 biosynthetic proteins [25]. Legend: Y indicates the presence of a highly conserved gene, and N indicates the absence of a similar gene.

**Gene**	**Locus tag**	**% putative peptide sequence identity**	***R. meliponini* D21B**	***R. nectarea***	***R. epipactidis***	***R. australiborealis***	***R. collisarenosi***
RibA, GTP cyclohydrolase-2	Ga0456434_10_ 123815_124408	79	Y	Y	Y	Y	Y
RibD, pyrimidine deaminase/riboflavin biosynthesis protein	Ga0456434_02_ 33435_34553	64	Y	Y	Y	Y	Y
RibH, 6,7-dimethyl-8-ribityllumazine synthase	Ga0456434_02_ 34632_35102	84	Y	Y	N	N	N
RibE, riboflavin synthase	Ga0456434_12_ 259559_260167	72	Y	Y	Y	Y	Y

Vitamin B_3 (Niacin)

Genes associated with niacin biosynthesis are not yet fully characterized in bacteria, although a set of genes that are upregulated in the absence of niacin have been identified as putative niacin biosynthetic genes in *Streptococcus pneumoniae* [26]. These genes include *fba, rex, gapN, niaX, pncB-nadE, pnuC, gap, spd-1824, nadC, adhE, and adhB2*. Only four of the abovementioned genes, *fba, pncB, nadE*, and *pnuC* have been identified across the five *Rosenbergiella* genomes (Table S17). The absence of a fully functional niacin-synthesizing pathway can be inferred from the inability of either *R. epipactidis* 2.1A or *R. meliponini* D21B to grow in minimal medium without niacin supplementation (Table S14).

Table S17: Vitamin B_3 biosynthetic genes in *R. meliponini* D21B and the four reported *Rosenbergiella* species with % of putative protein sequence identity to the *Streptococcus pneumonia* vitamin B_2 biosynthetic proteins [26]. Legend: Y indicates the presence of a highly conserved gene, and N its absence.

**Gene**	**Locus Tag**	**% putative peptide sequence identity**	***R. meliponini* D21B**	***R. nectarea***	***R. epipactidis***	***R. australiborealis***	***R. collisarenosi***
fbaA, fructose-bisphosphate aldolase	Ga0456434_04_213367_214446	26	Y	Y	Y	Y	Y
rex, a redox-sensitive transcriptional regulator			N	N	N	N	N
pncB, a nicotinate phosphoribosyltransferase	Ga0456434_07_33276_34481	26	Y	Y	Y	Y	Y
nadE, a NAD^+ synthetase	Ga0456434_12_228069_228896	66	Y	Y	Y	Y	Y
gapA, a glyceraldehyde-3-phosphate dehydrogenase	Ga0456434_10_189930_190928	51	Y	Y	Y	Y	Y
gapN, a glyceraldehyde-3-phosphate dehydrogenase	Ga0456434_10_48014_49480	32	N	N	N	N	N

**Gene**	**Locus Tag**	**% putative peptide sequence identity**	***R. meliponini* D21B**	***R. nectarea***	***R. epipactidis***	***R. australiborealis***	***R. collisarenosi***
adhE, an alcohol dehydrogenase	Ga0456434_12_63166_63525	33	N	N	N	N	N
adhB2, a zinc-containing alcohol dehydrogenase			N	N	N	N	N
NiaX, a niacin ECF transporter			N	N	N	N	N
PnuC, a ribosyl nicotinamide transporter	Ga0456434_07_145749_146465	22	Y	Y	Y	Y	Y
NadC, a nicotinate-nucleotide pyrophosphorylase			N	N	N	N	N

Vitamin B_5 (Pantothenic acid)

The enzymes associated with pantothenic acid biosynthesis are *ilvD*, *panB*, *panE*, *panD* and *panC* [27,28]. PanM regulates PanD activity. The genes *ilvD*, *panB*, *panE*, and *panC* are conserved in all five sequenced *Rosenbergiella* strains, but no homologues were found for *panD* and *panM* (Table S18). Nevertheless, both *R. meliponini* D21B and *R. epipactidis* 2.1A grew well in minimal medium without supplemented pantothenic acid (Table S14), indicating that they can synthesize this vitamin.

Table S**18:** Vitamin B_5 biosynthetic genes in *R. meliponini* D21B and the four reported *Rosenbergiella* species with % of putative protein sequence identity to the *E. coli* K12 vitamin B_5 biosynthetic proteins [27-29]. Legend: Y indicates the presence of a highly conserved gene, and N indicates the absence of a similar gene.

**Gene**	**Locus Tag**	**% Putative peptide sequence identity**	***R. meliponini* D21B**	***R. nectarea***	***R. epipactidis***	***R. australiborealis***	***R. collisarenosi***
ilvD, dihydroxy-acid dehydratase	Ga0456434_13_6468_8318	88	Y	Y	Y	Y	Y
PanB, 3-methyl-2-oxobutanoate hydroxymethyltransferase	Ga0456434_06_483760_484557	69	Y	Y	Y	Y	Y
PanE, 2-dehydropantoate 2-reductase	Ga0456434_02_41879_42796	49	Y	Y	Y	Y	Y
PanD, aspartate 1-decarboxylase			N	N	N	N	N
PanM, PanC regulator			N	N	N	N	N
PanC, pantothenate synthetase	Ga0456434_06_482901_483749	60	Y	Y	Y	Y	Y

Vitamin B_6 (pyridoxine)

De novo biosynthesis of pyridoxine in *E. coli* K12 involves five enzymes: D-erythrose-4-phosphate dehydrogenase (epd), erythronate-4-phosphate dehydrogenase (PdxB), phosphoserine aminotransferase (SerC), 4-hydroxythreonine-4-phosphate dehydrogenase (PdxA), and pyridoxine 5'-phosphate synthase (PdxJ) [30]. All corresponding genes are highly conserved across the *Rosenbergiella* strains examined (Table S19).

Table S**19:** Vitamin B_6 biosynthetic genes in *R. meliponini* D21B the four reported *Rosenbergiella* species with % of putative protein sequence identity to the *E. coli* K12 vitamin B_6 biosynthetic proteins. [30] Y indicates the presence of a highly conserved gene, and N indicates the absence of a similar gene.

**Gene**	**Locus tag**	**% putative peptide sequence identity**	***R. meliponini* D21B**	***R. nectarea***	***R. epipactidis***	***R. australiborealis***	***R. collisarenosi***
epd, D-erythrose-4-phosphate dehydrogenase	Ga0456434_04_215738_216766	78	Y	Y	Y	Y	Y
PdxB, erythronate-4-phosphate dehydrogenase	Ga0456434_01_228078_229208	64	Y	Y	Y	Y	Y
SerC, aminotransferase	Ga0456434_07_62623_63708	74	Y	Y	Y	Y	Y
PdxA, 4-hydroxythreonine-4-phosphate dehydrogenase	Ga0456434_06_389457_390449	75	Y	Y	Y	Y	Y
PdxJ, pyridoxine 5'-phosphate synthase	Ga0456434_01_32948_33679	74	Y	Y	Y	Y	Y

Vitamin B_7 (biotin)

The biotin biosynthesis pathway differs in various bacteria. However, pimelate is a common precursor [31]. The origin of pimelate remains somewhat unclear, But most bacteria have a passive permease that allows the uptake of this dicarboxylic acid into the cell [31]. Pimelate is converted to a pimeloyl-CoA by pimeloyl-CoA synthetase, the gene product of *pauA* in *Pseudomonas mendocina* 35 [32], and *bioW* in *Bacillus sphaericus* [31]. BioW is expressed by the *bioXWF* cluster, where BioX was thought to be a permease, while BioF was identified as the 8-amino-7-oxononanoate synthase. BioWF is a bifunctional enzyme found in some bacterial species including *Corynebacterium kroppenstedtii* [33], which catalyses both the generation of pimeloyl-CoA from pimelate, as well as the decarboxylative condensation of pimeloyl-CoA into (*S*)-8-amino-7-oxononanoate. BioF, which is found in *E.coli* as well as in all *Rosenbergiella* type strains, uses pimeloyl-acyl carrier protein rather than pimeloyl-CoA to produce 8-amino-7-oxononanoate [34,35].

The remaining steps of biotin biosynthesis are catalysed by BioA, BioD2 and BioB [36]. BioA is an adenosylmethionine-8-amino-7-oxononanoate aminotransferase, which produces *S*-adenosyl-4-methylsulfanyl-2-oxobutanoate from (*S*)-8-amino-7-oxononanoate and is conserved in all *Rosenbergiella* strains [37]. BioD2 is an ATP-dependent dethiobiotin synthetase. It was not found in any *Rosenbergiella* strain, but two genes with 50% and 63% protein sequence identity to *bioD* detected in *Mycobacterium tuberculosis* (strain ATCC 25618 / H37Rv) [38] and *bioD1* detected in Escherichia coli (strain K12) [39] respectively, which are also ATP-dependent detiobiotin enzymes, were found in the *R. meliponini* D21B and *R. nectarea* 8N4 genomes (Table S20). BioD is also found in the genomes of *R. epipactidis* 2.1A, *R. australiborealis* CdVSA20.1 and *R. collisarenosi* 8.8A.

Table S**20:** Vitamin B_7 biosynthetic genes in *R. meliponini* D21B and the four reported *Rosenbergiella* species with % of putative protein sequence identity to the *E. coli* K12 vitamin B_7 biosynthetic proteins. Y indicates the presence of a highly conserved gene and N indicates the absence of a similar gene.

**Gene**	**Locus tag**	**% putative peptide sequence identity**	***R. meliponini* D21B**	***R. nectarea***	***R. epipactidis***	***R. australiborealis***	***R. collisarenosi***
PauA, pimeloyl-CoA synthetase			N	N	N	N	N
BioW, pimeloyl-CoA synthetase (*Lysinibacillus spericus*)			N	N	N	N	N
BioF, 8-amino-7-oxononanoate synthase	Ga0456434_07_132605_133753	56	Y	Y	Y	Y	Y
BioA, adenosylmethionine-8-amino-7-oxononanoate aminotransferase	Ga0456434_07_134866_136155	66	Y	Y	Y	Y	Y
BioD1, ATP-dependent dethiobiotin synthetase BioD 1	Ga0456434_07_131171_131854	63	N	N	N	N	N
BioD, ATP-dependent dethiobiotin synthetase BioD	Ga0456434_10_74909_75586	50	Y	Y	Y	Y	Y
BioD 2, ATP-dependent dethiobiotin synthetase			N	N	N	N	N
BioB, biotin synthase	Ga0456434_07_133753_134784	80	Y	Y	Y	Y	Y

Vitamin B_9 (folate)

The folate biosynthesis in *E. coli* requires FolE, FolK, FolP and FolC [40-43]. These genes are well conserved across all *Rosenbergiella* type strains and *in R. meliponini* D21B, except for the apparent absence of a gene corresponding to *folP* in *R. epipactidis* 2.1A. Neither *R. meliponini* D21B nor *R. epipactidis* 2.1A required folate to grow in minimal medium (Table S14).

Table S21: Vitamin B_9 biosynthetic genes in *R. meliponini* D21B and the four reported *Rosenbergiella* species with % of putative protein sequence identity to the *E. coli* K12 vitamin B_9 biosynthetic proteins [40-43]. Legend: Y indicates the presence of a highly conserved gene and N indicates the absence of a similar gene.

**Gene**	**Locus tag**	**% Putative peptide sequence identity**	***R. meliponini* D21B**	***R. nectarea***	***R. epipactidis***	***R. australiborealis***	***R. collisarenosi***
FolE, GTP cyclohydrolase	Ga0456434_10_405220_405885	83	Y	Y	Y	Y	Y
FolK, protein Kinase, 2-amino-4-hydroxy-6-hydroxymethyl-dihydropteridine pyrophosphokinase	Ga0456434_06_484640_485119	56	Y	Y	Y	Y	Y
FolP, dihydropteroate synthase	Ga0456434_06_100429_101262	74	Y	Y	Y	Y	Y
FolC, dihydrofolate synthase/folylpolyglutamate synthase	Ga0456434_01_232901_234154	65	Y	Y	Y	Y	Y
YgfZ, folate-binding protein	Ga0456434_04_194684_195649	57	Y	Y	Y	Y	Y

Vitamin B_{10} (4-aminobenzoic acid)

4-Aminobenzoic acid synthesis in *E. coli* is catalysed by PabA, PabB, and PabC, using chorismate as precursor [44,45] producing 4-aminobenzoic acid for the folate biosynthesis pathway. All *Rosenbergiella* strains have genes with high level of conservation to *E. coli* K12 *pabB* and *pabC.*

The pabA gene is not well conserved, but it can be inferred that the catalytic activity of this enzyme can be met by another gene because both *R. meliponini* D21B and *R. epipactidis* 2.1A grow in absence of 4-aminobenzoic acid in the minimal (Tables A14, A22).

Table S**22:** Vitamin B_{10} biosynthetic genes in *R. meliponini* D21B and the four reported *Rosenbergiella* species with % of putative protein sequence identity to the *E. coli* K12 vitamin B_{10} biosynthetic proteins. [44,45] Y indicates the presence of a highly conserved gene, N indicates the absence of a similar gene.

**Gene**	**Locus tag**	**Putative peptide sequence identity in %**	***R. meliponini* D21B**	***R. nectarea***	***R. epipactidis***	***R. australiborealis***	***R. collisarenosi***
PabA, aminodeoxychorismate synthase component 2	Ga0456434_05_140367_140942	66	Y	Y	Y	Y	Y
PabB, aminodeoxychorismate synthase component 1	Ga0456434_10_209006_210379	61	Y	Y	Y	Y	Y
PabC, aminodeoxychorismate lyase	Ga0456434_12_115738_116547	52	Y	Y	Y	Y	Y

Vitamin B_{12} (cobalamin)

Vitamin B12 (cobalamin) is a cofactor for methyltransferase enzymes [46]. It is synthesized by some bacteria in three steps, while others acquire it [46,47]. The *hemABCDL* operon codes for the synthesis of uroporphyrinogen III, which is a precursor for the production of heme, shiroheme, as well as cobalamin, for example in *Salmonella typhimunum* [48]. The *Cbi* operon from *Bacillus megaterium* that has been shown to synthesize cobalamin under anaerobic conditions [49]. The *cob* operon from *S. typhimurium* biosynthesises cobalamin under aerobic conditions [50-53]. All five *Rosenbergiella* genomes comprise the *hemABCDL* operon that produces uroporphyrinogen III. However, all *Rosenbergiella* genomes lack *cbi* operon or *cob* operon genes that code for enzymes to produce cobalamin. Cobalamin salvage pathway genes have been identified in *S. typhimunum* [53], however, these genes were not identified in any of the *Rosenbergiella* genomes examined (Table S23). *R. meliponini* D21B and *R. epipactidis* 2.1A grew well in minimal medium lacking vitamin B_{12}. However, the presence of a B_{12}-independent methyltransferase in the genome of *R. meliponini* D21B (Table S23) may mean that cobalamin is not an essential cofactor.

Table S23: Vitamin B_{12} biosynthetic genes in *R. meliponini* D21B and the four reported *Rosenbergiella* species with % of putative protein sequence identity to the *S. typhimunum* vitamin B_{12} biosynthetic proteins. Y indicates the presence of a highly conserved gene, N indicates the absence of a similar gene, and U indicates 'uncertain', where the possible function of the gene product has been observed, but genomic annotation have not revealed a potential gene.

**Gene**	**Locus Tag**	**Putative peptide sequence identity in %**	***R. meliponini* D21B**	***R. nectarea***	***R. epipactidis***	***R. australiborealis***	***R. collisarenosi***
hemABCD	Ga0456434_01_ 204964-207227	76-99	Y	Y	Y	Y	Y
hemL (glutamate-1-semialdehyde 2,1-aminomutase)	Ga0456434_06_ 494670_495953	80	Y	Y	Y	Y	Y
cbi operon genes (*cbiABCDETFGHJKLMNOQP*)			N	N	N	N	N
cob operon genes			N	N	N	N	N
**Cobalamin salvage pathway genes**							
CysG, siroheme synthase	Ga0456434_04_ 49147_50562	94	Y	Y	Y	N	Y
btuB, Vit B12 transporter BtuB (*Escherichia hermannii*) [54]	Ga0456434_08_ 81185_83302	25	U	N	N	N	N
btuF, YadT, Vitamin B_{12}-binding protein	Ga0456434_18_ 65337_66170	27	U	N	N	N	N
butC, Vit B12 import system permease protein	Ga0456434_18_ 66154_67158	39	U	N	N	N	N
BtuD, Vitamin B12 import ATP-binding protein	Ga0456434_18_ 67151_67930	33	U	N	N	N	N
Methionine synthase (MetH) (*E. coli* K12)			N	N	N	N	N
Methionine synthase (B_{12}-independent) (*R. nectarea* 8N4)	Ga0456434_06_16 4293_165324	99	Y	Y	Y	Y	Y
Methylmalonyl-CoA mutase (scpA)			N	N	N	N	N

Vitamin K (menaquinone) synthesis genes as described in *Bacillus subtilis* [55,56] appear to be missing in all *Rosenbergiella* strains, except for ubiquinone/menaquinone biosynthesis C-methyltransferase (Table S24).

Table S24: Potential vitamin K biosynthetic genes in *R. meliponini* D21B and the four reported *Rosenbergiella* species in comparison to their annotated counterparts from *E. coli* K12 strain. Y indicates the presence of a highly conserved gene, N indicates the absence of a similar gene.

**Gene**	**Locus tag**	**Putative peptide sequence identity in %**	***R. meliponini* D21B**	***R. nectarea***	***R. epipactidis***	***R. australiborealis***	***R. collisarenosi***
menB, 1,4-dihydroxy-2-naphthoyl-CoA synthase			N	N	N	N	N
menC, o-succinylbenzoate synthase			N	N	N	N	N
menD, 2-succinyl-5-enolpyruvyl-6-hydroxy-3-cyclohexene-1-carboxylate synthase			N	N	N	N	N
menE, 2-succinylbenzoate--CoA ligase			N	N	N	N	N
menF, Isochorismate synthase MenF			N	N	N	N	N
menA, 1,4-dihydroxy-2-naphthoate octaprenyltransferase			N	N	N	N	N
ubiE, ubiquinone/menaquinone biosynthesis C-methyltransferase	Ga0456434_13_154840_155595	85	Y	Y	Y	Y	Y
ubiquinone biosynthesis protein UbiJ	Ga0456434_13_155595_156200	46	Y	Y	N	N	N

Biosynthesis of 2-phenylethanol

Phenylalanine, which is precursor in the production of 2-phenylethanol, can be synthesized by all *Rosenbergiella* strains via the shikimate pathway (Table S25) [57]. 2-Phenylethanol biosynthesis in *Proteus mirabilis* (order Enterobacterales) has been investigated in detail [58]. Eleven enzymes, including five aminotransferases, two decarboxylases and four alcohol dehydrogenases are involved in 2-phenylethanol synthesis via three different biochemical pathways: the amino acid deaminase (AAD) pathway, the Ehrlich pathway, and the aromatic amino acid decarboxylase (AADC) pathway.

Amino acid deaminase (AAD) pathway

The amino acid deaminase (AAD) pathway utilizes an L-amino acid deaminase (*Pma* and *Pm1*), an α-keto acid decarboxylase (KDC) and an alcohol dehydrogenase (ADH) for the conversion of L-phenylalanine into 2-phenylethanol (Figure A10). The *R. meliponini* D21B genome contains a gene with 70 % peptide sequence identity to *P. mirabilis* L-amino acid deaminase. An α-keto acid decarboxylase (KDC) converts phenylpyruvate (PPA) to phenyl-acetaldehyde (PAAL). However, the *R. meliponini* D21B draft genome lacks any gene with high homology to the *P. mirabilis* α-keto acid decarboxylase. In the final step an alcohol dehydrogenase (ADH) converts 2-phenylacetaldehyde to 2-phenylethanol. *R. meliponini* D21B and all other four *Rosenbergiella* species contain at least one gene that encodes for a class 4 alcohol dehydrogenase (ADH) (Table S26).

The Ehrlich pathway

The Ehrlich pathway converts L-phenylalanine into 2-phenylethanol in three steps: transamination of L-phenylalanine, decarboxylation of L-phenylalanine, and reduction of the aldehyde moiety [59-61]. L-phenylalanine is converted to phenylpyruvic acid (PAA) by the aromatic amino acid transaminase (AAAT) type 1 and type 2 (AAAT-2), for both there are candidate genes in *R. meliponini* D21B. Then the PAA is converted to phenylacetaldehyde (PAAL) by keto acid decarboxylases. The *R. meliponini* D21B draft genome comprises a gene with 36 % peptide sequence identity to the *P. mirabilis* keto acid decarboxylase. The last step of the Ehrlich pathway is the reduction of phenylacetaldehyde into 2-phenylethanol using ADH, which appears to be available to all *Rosenbergiella* strains isolated from *T. carbonaria*.

Aromatic amino acid decarboxylase (AADC) pathway

The third, possible pathway that 2-phenylethanol is synthesized is via the aromatic amino acid decarboxylase (AADC) pathway. In *P. mirabilis*, the initial conversion of L-phenylalanine to 2-phenylethylamine is catalysed by a pyridoxal-dependent decarboxylase. The closest match to this gene region in *R. meliponini* D21B only shares a 30 % peptide sequence identity with the

P. mirabilis counterpart, making it unlikely that this gene encodes an enzyme with the similar function. Subsequently in the pathway, L-aromatic amino acid transferase (AAAT) converts the 2-phenylethylamine into the aldehyde form, 2-phenylacetaldehyde. However, it was noted that the catalytic activity of AAATs towards 2-PEA was about 1000 fold less in comparison to its activity towards L-phenylalanine, indicating that 2-phenylalanine is the preferred substrate (as discussed above in the amino acid deaminase (AAD) pathway). The final step, as in the pathways discussed before, is the reduction of 2-phenylacetaldehyde into 2-phenylethanol by an alcohol dehydrogenase (ADH). Genes that could encode a type 4 ADH are present in all five *Rosenbergiella* genomes examined (Table S26). However, while not all *Rosenbergiella* examined here produced 2-phenylethanol, 2-phenylacetic acid was a ubiquitous product (Figure 8). This most likely arises from the oxidation of 2-phenylacetaldehyde rather than reduction. The *R. meliponini* D21B draft genome shows the presence of two aldehyde dehydrogenase encoding genes (Table S26).

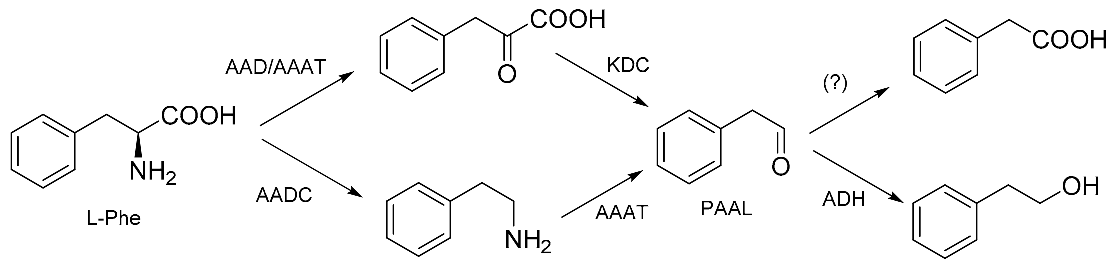

Figure A10: Biochemical pathways for the synthesis of 2-phenylethanol and 2-phenylacetic acid, based on those described for *Proteus mirabilis* [58]. All pathways lead to 2-phenyl-acetaldehyde (PAAL) as the preultimate intermediate. AAT: L-amino acid deaminase, AAAT: L-aromatic amino acid transferase, KDC: α-keto acid decarboxylase, ADH: Alcohol dehydrogenase,and (?): unknown, step not described for *P. mirabilis*. Possibly catalysed by aldehyde dehydrogenase.

Table S25: Conservation of the shikimate pathway genes in *R. meliponini* D21B and the four reported *Rosenbergiella* species with % of putative protein sequence identity to the *E. coli* K12 shikimate pathway biosynthetic proteins.

**Gene**	**Locus tag**	**Peptide sequence identity in %**	***R. meliponini* D21B**	***R. nectarea***	***R. epipactidis***	***R. australiborealis***	***R. collisarenosi***
aroG/DAHP, 3-deoxy-D-arabinoheptulosonate-7-phosphate synthase	Ga0456434_12_222768_223622	24 %	Y	Y	Y	Y	Y
arob/ DHQ synthase, 2,3-dehydroquinate synthase	Ga0456434_05_132683_133765	77 %	Y	Y	Y	Y	Y
aroC/DH-quinase, 3,3-dehydroquinate dehydratase	Ga0456434_05_132683_133765	77 %	Y	Y	Y	Y	Y
aroE/ shikimate_DH,shikimate dehydrogenase	Ga0456434_03_26019_26840	55 %	Y	Y	Y	Y	Y
aroK/SKI, shikimate kinase	Ga0456434_05_132122_132643	93 %	Y	Y	Y	Y	Y
aroA/ EPSP, 5-enolpyruvyl-shikimate-3-phosphate synthase	Ga0456434_07_61285_62577	79 %	Y	Y	Y	Y	Y
aroC, chorismate synthase	Ga0456434_01_215097_216176	84 %	Y	Y	Y	Y	Y

Table S26: Conservation of the Ehrlich pathway genes in *R. meliponini* D21B and the four reported *Rosenbergiella* species with % of putative protein sequence identity to 2-phenylethanol synthesising genes of *P. mirabilis*.

**Gene**	**Locus tag**	**Peptide sequence identity in %**	***R. meliponini* D21B**	***R. nectarea***	***R. epipactidis***	***R. australiborealis***	***R. collisarenosi***
Pma (WP_004246855.1)	Ga0456434_08_83571_84992	54	Y	N	N	N	N
***Pm1* (WP_004248621.1)**	**Ga0456434_08_83571_84992**	**70**	**Y**	**N**	**N**	**N**	**N**
KDC: α-keto acid decarboxylase (WP_012367760.1)	Ga0456434_12_167796_169478	36	Y	Y	Y	Y	Y
ADH: alcohol dehydrogenase IV, ADH-4	Ga0456434_10_332502_333611	83	Y	Y	Y	Y	Y
AAAT: L-aromatic amino acid transaminase, AAAT-1 (WP_012368490.1)	Ga0456434_18_20030_21214	61	Y	Y	Y	Y	Y
L-aromatic amino acid transaminase, AAAT-2 (WP_012367734.1)	Ga0456434_07_37464_38657	65	Y	Y	Y	Y	Y
pyridoxal-dependent decarboxylase (WP_017628132.1)	Ga0456434_08_74680_76209	30	Y	Y	Y	N	Y

**Gene**	**Locus tag**	**Peptide sequence identity in %**	***R. meliponini* D21B**	***R. nectarea***	***R. epipactidis***	***R. australiborealis***	***R. collisarenosi***
aldehyde dehydrogenase (*R. epipactidis*) (WP_214216111.1)	Ga0456434_12_185485_187005	99	Y	Y	Y	Y	Y
aldehyde dehydrogenase (*R. epipactidis*) (WP_214216226.1)	Ga0456434_10_48014_49480	99	Y	N	N	N	N

Resistance to environmental challenges

Antibiotic resistance

Antibiotic resistance mechanisms in bacteria involve restricting uptake, inactivation, or the modification of antimicrobial targets, as well as removal of xenobiotics [62]. IMG annotation and manual searches have not succeeded in detecting any genes in the *R. meliponini* D21B genome that are associated with the first three resistance mechanisms, however genes encoding for at least three distinct multidrug efflux pumps were identified (Table S27). KEGG Orthology (KO) database predicted the first gene (locus tags: Ga0456434_04_310767-Ga0456434_04_313901) to be homologous to *acrB*, *mexB*, *adeJ*, *smeE*, *mtrD*, *cmeB* multidrug efflux pumps. The second gene (Ga0456434_04_127933_ Ga0456434_04_131124) and the third gene (Ga0456434_04_124832- Ga0456434_04_127936) are predicted to encode for MdtB and MdtC, respectively. The mdtABC complex is a known *E. coli* efflux pump, and it confers resistance to novobiocin and deoxycholate [63,64]. A fourth gene (locus tags: Ga0456434_02_68155-Ga0456434_02_71271) is predicted to encode for the pore domain of the multidrug efflux transporter AcrB, which confers resistance to a wide variety of antibiotics in *E. coli* [65].

Table S27: Genes associated with multidrug efflux pumps from *R. meliponini* D21B and the four reported *Rosenbergiella* type strains

R. meliponini D21B	**Locus tag**	***R. nectarea***	***R. epipactidis***	***R. australiborealis***	***R. collisarenosi***
membrane fusion protein (multidrug efflux system)	Ga0456434_02_71300-72478<br>Ga0456434_04_131124-13235<br>Ga0456434_04_313917-315047	Y	Y	Y	Y
multidrug efflux pump	Ga0456434_02_68155_71271<br>Ga0456434_04_124832_127936<br>Ga0456434_04_127933_131124<br>Ga0456434_04_310767_313901	Y	Y	Y	Y
multidrug efflux system membrane fusion protein	Ga0456434_04_131124_132350<br>Ga0456434_04_309374_310774	Y	Y	Y	Y

Genes associated with virulence of *R. meliponini* D21B

The genome of *R. meliponini* D21B contains several hemolysin-associated genes, as well as genes that protect against osmotic, oxidative and acid stress, which are also present in the *R. nectarea* 8N4 genome. The *R. meliponini* D21B genome contains many genes that encode fimbrial associated proteins, which are lacking in the *R. nectarea* 8N4 genome (Table S28). In contrast to *R. nectarea* 8N4, *R. meliponini* D21B does not comprise genes associated with pectin lyase or the P2-like prophage.

Table S28: Virulence factor genes in *R. meliponini* D21B and other four *Rosenbergiella* type strains.

**Virulence factor**	**Locus tag**	***R. meliponini D21B***	***R. nectarea***	***R. epipactidis***	***R. australiborealis***	***R. collisarenosi***
Pectin lyase		N	Y	N	N	N
Pectate lyase		N	Y	N	N	N
P2-like prophage		N	Y	N	N	N
Elongation factor P hydroxylase	Ga0456434_01_ 217837-218382	Y	Y	Y	Y	Y
Adhesion						
Filamentous hemagglutinin	Ga0456434_04_ 141414-149411	Y	Y	Y	Y	Y
Fimbria synthesis						
Major type-1 subunit fimbrin (pilin, fimA)	Ga0456434_04_ 27862-28410	Y	Y	Y	Y	N
**Hemolysin**						
Hemolysin, contains CBS domains		N	N	N	N	N
ShlB/FhaC/HecB family hemolysin secretion/activation protein	Ga0456434_04_ 149422_151173	Y	Y	Y	Y	Y
Multispecies hemolysin family protein		N	Y	N	N	Y
Hemolysin III family protein	Ga0456434_04_ 195720_196373	Y	Y	Y	Y	Y
Hemolysin expression modulator Hha	Ga0456434_02_ 67237_67461	Y	Y	Y	Y	Y

**Virulence factor**	**Locus tag**	***R. meliponini D21B***	***R. nectarea***	***R. epipactidis***	***R. australiborealis***	***R. collisarenosi***
**Motility**						
cyclo-di-GMP-binding flagellar-brake protein YcgR, contains PilZNR and PilZ domains		N	N	N	N	N
flagellum synthesis protein FlgN	Ga0456434_12_ 92936_93364	Y	Y	Y	Y	Y
negative regulator of flagellin synthesis FlgM	Ga0456434_12_ 93354_93656	Y	Y	Y	Y	Y
flagellum basal body P-ring formation protein FlgA	Ga0456434_12_ 93748_94392	Y	Y	Y	Y	Y
flagellar basal-body rod protein FlgB	Ga0456434_12_ 94542_94952	Y	Y	Y	Y	Y
flagellar basal-body rod protein FlgC	Ga0456434_12_ 94966_95364	Y	Y	Y	Y	Y
flagellar basal-body rod modification protein FlgD	Ga0456434_12_ 95378_96046	Y	Y	Y	Y	Y
flagellar hook protein FlgE	Ga0456434_12_ 96063_97265	Y	Y	Y	Y	Y
flagellar basal-body rod protein FlgF	Ga0456434_12_ 97281_98042	Y	Y	Y	Y	Y
flagellar basal-body rod protein FlgG	Ga0456434_12_ 98055_98837	Y	Y	Y	Y	Y
flagellar L-ring protein precursor FlgH	Ga0456434_12_ 98885_99580	Y	Y	Y	Y	Y
flagellar P-ring protein precursor FlgI	Ga0456434_12_ 99593_100693	Y	Y	Y	Y	Y
flagellar protein FlgJ	Ga0456434_12_ 100693_101601	Y	Y	Y	Y	Y
flagellar hook-associated protein 1 FlgK	Ga0456434_12_ 101698_103332	Y	Y	Y	Y	Y
flagellar hook-associated protein 3 FlgL	Ga0456434_12_ 103354_104316	Y	Y	Y	Y	Y
MAF protein (musculoaponeurotic fibrosarcoma), a transcription factor	Ga0456434_12_ 108841_109428	Y	Y	Y	Y	Y

Acid stress responses

In bacteria, acid stress usually induces changes in cell membrane integrity and fluidity, pH homeostasis, metabolic regulation and macromolecular repair [66]. The genome of *R. meliponini* D21B comprises two monovalent cation antiporters of the CPA2 and NhaA families, as well as a potassium efflux system protein KefB, all of which are responsible for maintaining the cellular pH [67-69]. Cyclopropanation of unsaturated phospholipids has been identified as an acid stress response [70]. All sequenced *Rosenbergiella* strains, including *R. meliponini* D21B, carry a cyclopropane-fatty-acyl-phospholipid synthase gene (Table S29).

Table S29: Genes associated with acid stress responses in *R. meliponini* D21B and the four reported *Rosenbergiella* species. Percentage peptide sequence identity for each gene is shown in comparison to the characterized gene from *E. coli* K12 extracted from UniProt (uniprot.org), unless an alternative source is specified.

**Genes associated with acid stress responses**	**Locus tag**	**Peptide sequence identity in %**	***R. meliponini* D21B**	***R. nectarea***	***R. epipactidis***	***R. australiborealis***	***R. collisarenosi***
Potassium efflux system, KefB	Ga0456434_02_ 82917-84608	75	Y	Y	Y	Y	Y
Glutathione-regulated potassium-efflux system protein, KefC	Ga0456434_05_ 151168-152979	42	Y	Y	Y	Y	Y
NhaA (Na+/H+ antiporter like domain)	Ga0456434_02_ 99635-100780	49	Y	Y	Y	Y	Y
cyclopropane-fatty-acyl-phospholipid synthase	Ga0456434_12_ 260203-261351	79	Y	Y	Y	Y	Y

Osmotic stress

Aquaporins are fundamental for the maintenance of the cellular osmotic balance by the transfer of water across the membrane. Well-known bacterial aquaporins, including aquaporin Z (AqpZ) from *B. cereus* are not found in any of the *Rosenbergiella* strains (Table S30). However, *E. coli* ProX is a glycerol uptake facilitator protein [71] that is closely related to aquaporin family proteins from many Enterobacteriaceae. It is found in *R. meliponini* D21B and all *Rosenbergiella* strains except in *R. epipactidis* 2.1A. ProX is part of the ProU ABC transporter complex that imports the osmoprotectant glycine betaine into the cells [71,72]. A gene annotated as OsmY, a possible osmoprotectant import permease protein, with 42 % peptide sequence identity to the *E. coli* K12 counterpart, was found in *R. meliponini* D21B. *R. epipactidis* 2.1A, *R. nectarea* 84N, *R. australiborealis* CdVSA20.1 and *R. collisarenosi* 8.8A have genes with 97 %, 85 %, 70 % and 77 % peptide sequence identity to OsmY, respectively.

Table S30: Genes associated with osmotic stress responses in *R. meliponini* D21B and the four reported *Rosenbergiella* species. Percentage peptide sequence identity is not shown (denoted 'n/a') when the product of the gene discussed, have been characterized in the IMG annotation process. Percentage peptide sequence identity for each gene is shown in comparison to the characterized gene from *E. coli* K12 extracted from UniProt (uniprot.org). Y indicates the presence of a highly conserved gene, N indicates the absence of a similar gene.

**Genes associated with osmotic stress responses**	**Locus tag**	**Peptide sequence identity in %**	***R. meliponini* D21B**	***R. nectarea***	***R. epipactidis***	***R. australiborealis***	***R. collisarenosi***
betA, choline dehydrogenase	Ga0456434_04_328863-330494	n/a	Y	Y	Y	Y	Y
Choline/glycine/proline-betaine transport protein	Ga0456434_06_333425-335455	n/a	Y	Y	Y	Y	Y
Betaine aldehyde dehydrogenase	Ga0456434_01_89572-91002	n/a	Y	Y	Y	Y	Y
MFS transporter, MHS family, proline/betaine transporter	Ga0456434_04_320581-322089	n/a	Y	Y	Y	Y	Y
Osmotically inducible protein OsmY,	Ga0456434_10_164806-165120	42	Y	Y	Y	Y	Y
Hyperosmotically inducible protein	Ga0456434_04_264467-265048	n/a	Y	Y	Y	Y	Y
Osmotically inducible lipoprotein OsmB	Ga0456434_10_119939-120157	67	Y	Y	Y	Y	Y
Osmotically inducible lipoprotein OsmC	Ga0456434_02_15500-15931	74	Y	Y	Y	Y	Y
Osmotically inducible lipoprotein OsmE	Ga0456434_12_230365-230706	53	Y	Y	Y	Y	Y

**Genes associated with osmotic stress responses**	**Locus tag**	**Peptide sequence identity in %**	***R. meliponini* D21B**	***R. nectarea***	***R. epipactidis***	***R. australiborealis***	***R. collisarenosi***
Miniconductance mechanosensitive channel (protection against hypoosmotic shock)	Ga0456434_06_416205-417473	62	Y	Y	Y	Y	Y
AqpZ, aquaporin Z			N	N	N	N	N
ProX, part of the ProU ABC transporter complex	Ga0456434_14_11021-11998	69	Y	Y	N	Y	Y
ABC transporter proteins	Ga0456434_03_2083-3222		Y	Y	Y	Y	Y

Oxidative stress responses

In bacteria, oxidative stress responses are mediated by either the peroxide stimulon or the superoxide stimulon, where each pathway is encoded by about 30 genes [73-76]. These systems are under the control of OxyR and SoxRS regulators, which are positive regulatory elements. OxyR regulates the peroxide stimulus, which includes gene elements such as *fur* [77], alkyl hydroperoxide reductase C and F [78], and Dps (DNA protection during starvation protein) [79] [80]. Most bacterial oxidative stress response elements examined, are conserved in *Rosenbergiella* strains consistently (Table S31), except for catalase peroxidase (katG), and alkyl hydroperoxide reductase C (AhpC), which was not found in the *R. meliponini* D21B genome. Data shown (bold and with asterix) for *ahpC*, were from *R. epipactidis* 2.1A, and the absence of this gene in the *R. meliponini* D21B genome may be due to missing genes in the draft genome, since in all other *Rosenbergiella* genomes *ahpC* is highly conserved with 86-87 % peptide sequence identity with its *E. coli* K12 counterpart.

Table S31: Genes associated with oxidative stress responses in *R. meliponini* D21B and the four reported *Rosenbergiella* species. Percentage peptide sequence identity for each gene is shown in comparison to the characterized gene from *E. coli* K12 extracted from UniProt (uniprot.org), unless an alternative source is specified.

	**Locus tag**	**% Peptide sequence identity**	***R. meliponini* D21B**	***R. nectarea***	***R. epipactidis***	***R. australiborealis***	***R. collisarenosi***
AhpC, peroxiredoxin (alkyl hydroperoxide reductase subunit C)	Ga0456434_01_ 130071_130538	86	Y	Y	Y	Y	Y
Thiol peroxidase (atypical 2-Cys peroxiredoxin)	Ga0456434_10_ 103805_104308	69	Y	Y	Y	Y	Y
OxyR, LysR family hydrogen peroxide-inducible transcriptional activator	Ga0456434_08_ 163537_164454	86	Y	Y	Y	Y	Y
SoxR, MerR family redox-sensitive transcriptional activator	Ga0456434_06_ 190171_190692	68	Y	Y	Y	Y	Y
SoxS, AraC family mar-sox-rob regulon transcriptional activator	Ga0456434_06_ 189710_190075	63	Y	Y	Y	Y	Y
**Peroxide stimulon**							
katG, catalase-peroxidase			N	N	N	N	N
gor, glutathione reductase			N	N	N	N	N
ahpC, alkyl hydroperoxide reductase C	Ga0456434_10_39 6448_39701	86		Y	Y	Y	Y

	Locus tag	% Peptide sequence identity	*R. meliponini* D21B	*R. nectarea*	*R. epipactidis*	*R. australiborealis*	*R. collisarenosi*
ahpF, Alkyl hydroperoxide reductase subunit F	Ga0456434_10_394817_396382	73	Y	Y	Y	Y	Y
oxyS (hydrogen peroxide-inducible genes activator) (Uniprot accession number: L7N677_MYCTU from *Mycobacterium tuberculosis* strain ATCC 25618 / H37Rv			N	N	N	N	N
dps, DNA protection during starvation protein	Ga0456434_07_103434_103937	77	Y	Y	Y	Y	Y
fur, ferric uptake regulation protein	Ga0456434_07_179067_179513	87	Y	N	N	Y	Y
grxA, glutaredoxin 1	Ga0456434_07_91735_91998	66	Y	N	Y	N	Y
**Superoxide stimulon**							
sodA, superoxide dismutase (Binds 1 Mn^{2+} ion per subunit)	Fe-Mn family superoxide dismutase	86	Y	Y	Y	Y	Y
nfo, endonuclease 4	Ga0456434_10_409600_410439	79	Y	Y	Y	Y	Y
zwf, glucose-6-phosphate 1-dehydrogenase	Ga0456434_10_232743_234230	84	Y	Y	Y	Y	Y

References

1. Gordon, R.E.; Smith, N.R.; Pang, C.H.-N.; Haynes, W.C. *The genus Bacillus*; Agricultural Research Service, U.S. Dept. of Agriculture : For sale by Supt. of Docs., U.S. G.P.O.: Washington, D.C., 1973; pp. vii, 283 p.
2. Atlas, R.M., ed. *Handbook of Microbiological Media*; Atlas, R.M., Ed.; CRC Press: 2010.
3. Marmont, L.S.; Whitfield, G.B.; Rich, J.D.; Yip, P.; Giesbrecht, L.B.; Stremick, C.A.; Whitney, J.C.; Parsek, M.R.; Harrison, J.J.; Howell, P.L. PelA and PelB proteins form a modification and secretion complex essential for Pel polysaccharide-dependent biofilm formation in *Pseudomonas aeruginosa*. *J Biol Chem* **2017**, *292*, 19411-19422, doi:10.1074/jbc.M117.812842.
4. Núñez-Montero, K.; Quezada-Solís, D.; Khalil, Z.G.; Capon, R.J.; Andreote, F.D.; Barrientos, L. Genomic and Metabolomic Analysis of Antarctic Bacteria Revealed Culture and Elicitation Conditions for the Production of Antimicrobial Compounds. *Biomolecules* **2020**, *10*, 673, doi:10.3390/biom10050673.
5. Thurnheer, T.; Cook, A.M.; Leisinger, T. Co-culture of defined bacteria to degrade seven sulfonated aromatic compounds: efficiency, rates adn phenotypic variations. *Appl Microbiol Biotechnol* **1988**, *29*, 605-609.
6. Pfennig, N. *Rhodocyclus purpureus* gen. nov. and sp. nov., a Ring-Shaped, Vitamin B_{12}-Requiring Member of the Family Rhodospirillaceae. *Int J Sys Evol Microbiol* **1978**, *28*, 283-288, doi:10.1099/00207713-28-2-283.
7. Alvarez-Perez, S.; de Vega, C.; Vanoirbeek, K.; Tsuji, K.; Jacquemyn, H.; Fukami, T.; Michiels, C.; Lievens, B. Phylogenomic analysis of the genus Rosenbergiella and description of Rosenbergiella gaditana sp. nov., Rosenbergiella metrosideri sp. nov., Rosenbergiella epipactidis subsp. epipactidis subsp. nov., Rosenbergiella epipactidis subsp. californiensis subsp. nov., Rosenbergiella epipactidis subsp. japonicus subsp. nov., Rosenbergiella nectarea subsp. nectarea subsp. nov. and Rosenbergiella nectarea subsp. apis subsp. nov., isolated from floral nectar and insects. *Int J Syst Evol Microbiol* **2023**, *73*, doi:10.1099/ijsem.0.005777.
8. Jain, C.; Rodriguez-R, L.M.; Phillippy, A.M.; Konstantinidis, K.T.; Aluru, S. High throughput ANI analysis of 90K prokaryotic genomes reveals clear species boundaries. *Nat Commun* **2018**, *9*, 5114-5114, doi:10.1038/s41467-018-07641-9.
9. Sulavik, M.C.; Houseweart, C.; Cramer, C.; Jiwani, N.; Murgolo, N.; Greene, J.; DiDomenico, B.; Shaw, K.J.; Miller, G.H.; Hare, R.; et al. Antibiotic susceptibility profiles of *Escherichia coli* strains lacking multidrug efflux pump genes. *Antimicrob Agents Chemother* **2001**, *45*, 1126-1136, doi:10.1128/aac.45.4.1126-1136.2001.
10. Tauch, A.; Kirchner, O.; Löffler, B.; Götker, S.; Pühler, A.; Kalinowski, J. Efficient Electrotransformation of *Corynebacterium diphtheriae* with a Mini-Replicon Derived from the *Corynebacterium glutamicum* Plasmid pGA1. *Curr Microbiol* **2002**, *45*, 362-367, doi:10.1007/s00284-002-3728-3.
11. Hooper, D.C.; Wolfson, J.S.; McHugh, G.L.; Swartz, M.D.; Tung, C.; Swartz, M.N. Elimination of plasmid pMG110 from *Escherichia coli* by novobiocin and other inhibitors of DNA gyrase. *Antimicrob Agents Chemother* **1984**, *25*, 586-590, doi:10.1128/AAC.25.5.586.
12. Halpern, M.; Fridman, S.; Atamna-Ismaeel, N.; Izhaki, I. *Rosenbergiella nectarea* gen. nov., sp. nov., in the family *Enterobacteriaceae,* isolated from floral nectar. *Int J Syst Evol Microbiol* **2013**, *63*, 4259-4265, doi:10.1099/ijs.0.052217-0.
13. Lenaerts, M.; Alvarez-Pérez, S.; de Vega, C.; Van Assche, A.; Johnson, S.D.; Willems, K.A.; Herrera, C.M.; Jacquemyn, H.; Lievens, B. *Rosenbergiella australoborealis* sp. nov., *Rosenbergiella collisarenosi* sp. nov. and *Rosenbergiella epipactidis* sp. nov.,

three novel bacterial species isolated from floral nectar. *Syst Appl Microbiol* **2014**, *37*, 402-411, doi:10.1016/j.syapm.2014.03.002.

14. Blin, K.; Shaw, S.; Steinke, K.; Villebro, R.; Ziemert, N.; Lee, S.Y.; Medema, M.H.; Weber, T. antiSMASH 5.0: updates to the secondary metabolite genome mining pipeline. *Nucleic Acids Res* **2019**, *47*, W81-W87, doi:10.1093/nar/gkz310.
15. Blin, K.; Shaw, S.; Kloosterman, A.M.; Charlop-Powers, Z.; van Wezel, G.P.; Medema, Marnix H.; Weber, T. antiSMASH 6.0: improving cluster detection and comparison capabilities. *Nucleic Acids Res* **2021**, *49*, W29-W35, doi:10.1093/nar/gkab335.
16. Barona-Gómez, F.; Wong, U.; Giannakopulos, A.E.; Derrick, P.J.; Challis, G.L. Identification of a cluster of genes that directs desferrioxamine biosynthesis in *Streptomyces coelicolor* M145. *J Am Chem Soc* **2004**, *126*, 16282-16283, doi:10.1021/ja045774k.
17. Raymond, K.N.; Dertz, E.A.; Kim, S.S. Enterobactin: an archetype for microbial iron transport. *Proc Natl Acad Sci U S A* **2003**, *100*, 3584-3588, doi:10.1073/pnas.0630018100.
18. Salomone-Stagni, M.; Bartho, J.D.; Polsinelli, I.; Bellini, D.; Walsh, M.A.; Demitri, N.; Benini, S. A complete structural characterization of the desferrioxamine E biosynthetic pathway from the fire blight pathogen *Erwinia amylovora*. *J Struct Biol* **2018**, *202*, 236-249, doi:10.1016/j.jsb.2018.02.002.
19. Smits, T.H.; Duffy, B. Genomics of iron acquisition in the plant pathogen *Erwinia amylovora*: insights in the biosynthetic pathway of the siderophore desferrioxamine E. *Arch Microbiol* **2011**, *193*, 693-699, doi:10.1007/s00203-011-0739-0.
20. Choi, O.; Kang, B.; Lee, Y.; Lee, Y.; Kim, J.-H. *Pantoea ananatis* carotenoid production confers toxoflavin tolerance and is regulated by Hfq ‐ controlled quorum sensing. *MicrobiologyOpen* **2021**, *10*, doi:10.1002/mbo3.1143.
21. Misawa, N.; Satomi, Y.; Kondo, K.; Yokoyama, A.; Kajiwara, S.; Saito, T.; Ohtani, T.; Miki, W. Structure and functional analysis of a marine bacterial carotenoid biosynthesis gene cluster and astaxanthin biosynthetic pathway proposed at the gene level. *J Bacteriol* **1995**, *177*, 6575-6584, doi:10.1128/jb.177.22.6575-6584.1995.
22. Rodionov, D.A.; Vitreschak, A.G.; Mironov, A.A.; Gelfand, M.S. Comparative Genomics of Thiamin Biosynthesis in Procaryotes: New Genes and Regulatory Mechanisms *J Biol Chem* **2002**, *277*, 48949-48959, doi:10.1074/jbc.M208965200.
23. Du, Q.; Wang, H.; Xie, J. Thiamin (vitamin B_1) biosynthesis and regulation: a rich source of antimicrobial drug targets? *Int J Biol Sci* **2011**, *7*, 41-52, doi:10.7150/ijbs.7.41.
24. Webb, E.; Claas, K.; Downs, D.M. Characterization of thiI, a new gene involved in thiazole biosynthesis in *Salmonella typhimurium*. *J Bacteriol* **1997**, *179*, 4399-4402, doi:10.1128/jb.179.13.4399-4402.1997.
25. García-Angulo, V.A. Overlapping riboflavin supply pathways in bacteria. *Crit Rev Microbiol* **2017**, *43*, 196-209, doi:10.1080/1040841X.2016.1192578.
26. Afzal, M.; Kuipers, O.P.; Shafeeq, S. Niacin-mediated Gene Expression and Role of NiaR as a Transcriptional Repressor of niaX, nadC, and pnuC in *Streptococcus pneumoniae*. *Front Cell Infect Microbiol* **2017**, *7*, 70-70, doi:10.3389/fcimb.2017.00070.
27. Leonardi, R.; Jackowski, S. Biosynthesis of Pantothenic Acid and Coenzyme A. *EcoSal Plus* **2007**, *2*, doi:10.1128/ecosalplus.3.6.3.4.
28. Zheng, R.; Blanchard, J.S. Kinetic and mechanistic analysis of the *E. coli* panE-encoded ketopantoate reductase. *Biochemistry* **2000**, *39*, 3708-3717, doi:10.1021/bi992676g.
29. Blattner, F.R.; Plunkett, G., 3rd; Bloch, C.A.; Perna, N.T.; Burland, V.; Riley, M.; Collado-Vides, J.; Glasner, J.D.; Rode, C.K.; Mayhew, G.F.; et al. The complete genome sequence of *Escherichia coli* K-12. *Science* **1997**, *277*, 1453-1462, doi:10.1126/science.277.5331.1453.

30. Laber, B.; Maurer, W.; Scharf, S.; Stepusin, K.; Schmidt, F.S. Vitamin B_6 biosynthesis: formation of pyridoxine 5'-phosphate from 4-(phosphohydroxy)-L-threonine and 1-deoxy-D-xylulose-5-phosphate by PdxA and PdxJ protein. *FEBS Lett* **1999**, *449*, 45-48, doi:10.1016/s0014-5793(99)00393-2.
31. Ploux, O.; Soularue, P.; Marquet, A.; Gloeckler, R.; Lemoine, Y. Investigation of the first step of biotin biosynthesis in *Bacillus sphaericus*. Purification and characterization of the pimeloyl-CoA synthase, and uptake of pimelate. *Biochem J* **1992**, *287 (Pt 3)*, 685-690, doi:10.1042/bj2870685.
32. Binieda, A.; Fuhrmann, M.; Lehner, B.; Rey-Berthod, C.; Frutiger-Hughes, S.; Hughes, G.; Shaw, N.M. Purification, characterization, DNA sequence and cloning of a pimeloyl-CoA synthetase from *Pseudomonas mendocina* 35. *Biochem J* **1999**, *340 (Pt 3)*, 793-801.
33. Tauch, A.; Schneider, J.; Szczepanowski, R.; Tilker, A.; Viehoever, P.; Gartemann, K.H.; Arnold, W.; Blom, J.; Brinkrolf, K.; Brune, I.; et al. Ultrafast pyrosequencing of *Corynebacterium kroppenstedtii* DSM44385 revealed insights into the physiology of a lipophilic corynebacterium that lacks mycolic acids. *J Biotechnol* **2008**, *136*, 22-30, doi:10.1016/j.jbiotec.2008.03.004.
34. Webster, S.P.; Alexeev, D.; Campopiano, D.J.; Watt, R.M.; Alexeeva, M.; Sawyer, L.; Baxter, R.L. Mechanism of 8-amino-7-oxononanoate synthase: spectroscopic, kinetic, and crystallographic studies. *Biochemistry* **2000**, *39*, 516-528, doi:10.1021/bi991620j.
35. Lin, S.; Hanson, R.E.; Cronan, J.E. Biotin synthesis begins by hijacking the fatty acid synthetic pathway. *Nat Chem Biol* **2010**, *6*, 682-688, doi:10.1038/nchembio.420.
36. Sanyal, I.; Cohen, G.; Flint, D.H. Biotin synthase: purification, characterization as a [2Fe-2S]cluster protein, and in vitro activity of the *Escherichia coli* bioB gene product. *Biochemistry* **1994**, *33*, 3625-3631, doi:10.1021/bi00178a020.
37. Stoner, G.L.; Eisenberg, M.A. Purification and properties of 7, 8-diaminopelargonic acid aminotransferase. *J Biol Chem* **1975**, *250*, 4029-4036.
38. Cole, S.T.; Brosch, R.; Parkhill, J.; Garnier, T.; Churcher, C.; Harris, D.; Gordon, S.V.; Eiglmeier, K.; Gas, S.; Barry, C.E., 3rd; et al. Deciphering the biology of *Mycobacterium tuberculosis* from the complete genome sequence. *Nature* **1998**, *393*, 537-544, doi:10.1038/31159.
39. Otsuka, A.J.; Buoncristiani, M.R.; Howard, P.K.; Flamm, J.; Johnson, C.; Yamamoto, R.; Uchida, K.; Cook, C.; Ruppert, J.; Matsuzaki, J. The *Escherichia coli* biotin biosynthetic enzyme sequences predicted from the nucleotide sequence of the bio operon. *J Biol Chem* **1988**, *263*, 19577-19585.
40. Sybesma, W.; Starrenburg, M.; Kleerebezem, M.; Mierau, I.; de Vos, W.M.; Hugenholtz, J. Increased production of folate by metabolic engineering of *Lactococcus lactis*. *Appl Environ Microbiol* **2003**, *69*, 3069-3076, doi:10.1128/AEM.69.6.3069-3076.2003.
41. Rebelo, J.; Auerbach, G.; Bader, G.; Bracher, A.; Nar, H.; Hösl, C.; Schramek, N.; Kaiser, J.; Bacher, A.; Huber, R.; et al. Biosynthesis of pteridines. Reaction mechanism of GTP cyclohydrolase I. *J Mol Biol* **2003**, *326*, 503-516, doi:10.1016/s0022-2836(02)01303-7.
42. Swedberg, G.; Castensson, S.; Sköld, O. Characterization of mutationally altered dihydropteroate synthase and its ability to form a sulfonamide-containing dihydrofolate analog. *J Bacteriol* **1979**, *137*, 129-136, doi:10.1128/jb.137.1.129-136.1979.
43. Bognar, A.L.; Osborne, C.; Shane, B.; Singer, S.C.; Ferone, R. Folylpoly-gamma-glutamate synthetase-dihydrofolate synthetase. Cloning and high expression of the *Escherichia coli* folC gene and purification and properties of the gene product. *J Biol Chem* **1985**, *260*, 5625-5630.
44. Huang, M.; Gibson, F. Biosynthesis of 4-aminobenzoate in *Escherichia coli*. *J Bacteriol* **1970**, *102*, 767-773, doi:10.1128/jb.102.3.767-773.1970.

45. Green, J.M.; Nichols, B.P. p-Aminobenzoate biosynthesis in *Escherichia coli*. Purification of aminodeoxychorismate lyase and cloning of pabC. *J Biol Chem* **1991**, *266*, 12971-12975.
46. Fang, H.; Kang, J.; Zhang, D. Microbial production of vitamin B12: a review and future perspectives. *Microbial Cell Factories* **2017**, *16*, 15, doi:10.1186/s12934-017-0631-y.
47. Cadieux, N.; Bradbeer, C.; Reeger-Schneider, E.; Köster, W.; Mohanty, A.K.; Wiener, M.C.; Kadner, R.J. Identification of the periplasmic cobalamin-binding protein BtuF of *Escherichia coli*. *J Bacteriol* **2002**, *184*, 706-717, doi:10.1128/jb.184.3.706-717.2002.
48. Xu, K.; Delling, J.; Elliott, T. The genes required for heme synthesis in *Salmonella typhimurium* include those encoding alternative functions for aerobic and anaerobic coproporphyrinogen oxidation. *J Bacteriol* **1992**, *174*, 3953-3963, doi:10.1128/jb.174.12.3953-3963.1992.
49. Raux, E.; Lanois, A.; Rambach, A.; Warren, M.J.; Thermes, C. Cobalamin (vitamin B12) biosynthesis: functional characterization of the Bacillus megaterium cbi genes required to convert uroporphyrinogen III into cobyrinic acid a,c-diamide. *Biochem J* **1998**, *335 (Pt 1)*, 167-173, doi:10.1042/bj3350167.
50. Raux, E.; Lanois, A.; Levillayer, F.; Warren, M.J.; Brody, E.; Rambach, A.; Thermes, C. *Salmonella typhimurium* cobalamin (vitamin B_{12}) biosynthetic genes: functional studies in *S. typhimurium* and *Escherichia coli*. *J Bacteriol* **1996**, *178*, 753-767, doi:10.1128/jb.178.3.753-767.1996.
51. Roth, J.R.; Lawrence, J.G.; Rubenfield, M.; Kieffer-Higgins, S.; Church, G.M. Characterization of the cobalamin (vitamin B_{12}) biosynthetic genes of *Salmonella typhimurium*. *J Bacteriol* **1993**, *175*, 3303-3316, doi:10.1128/jb.175.11.3303-3316.1993.
52. Chen, P.; Ailion, M.; Weyand, N.; Roth, J. The end of the cob operon: evidence that the last gene (cobT) catalyzes synthesis of the lower ligand of vitamin B12, dimethylbenzimidazole. *J Bacteriol* **1995**, *177*, 1461-1469, doi:10.1128/jb.177.6.1461-1469.1995.
53. Lawrence, J.G.; Roth, J.R. Evolution of coenzyme B_{12} synthesis among enteric bacteria: evidence for loss and reacquisition of a multigene complex. *Genetics* **1996**, *142*, 11-24, doi:10.1093/genetics/142.1.11.
54. The UniProt, C. UniProt: the universal protein knowledgebase in 2021. *Nucleic Acids Res* **2021**, *49*, D480-D489, doi:10.1093/nar/gkaa1100.
55. Driscoll, J.R.; Taber, H.W. Sequence organization and regulation of the *Bacillus subtilis* menBE operon. *J Bacteriol* **1992**, *174*, 5063-5071, doi:10.1128/jb.174.15.5063-5071.1992.
56. Bentley, R.; Meganathan, R. Biosynthesis of vitamin K (menaquinone) in bacteria. *Microbiol Rev* **1982**, *46*, 241-280, doi:10.1128/mr.46.3.241-280.1982.
57. Herrmann, K.M.; Weaver, L.M. The Shikimate Pathway. *Annu Rev Plant Physiol Plant Mol Biol* **1999**, *50*, 473-503, doi:10.1146/annurev.arplant.50.1.473.
58. Liu, J.; Bai, Y.; Fan, T.P.; Zheng, X.; Cai, Y. Unveiling the Multipath Biosynthesis Mechanism of 2-Phenylethanol in *Proteus mirabilis*. *J Agric Food Chem* **2020**, *68*, 7684-7690, doi:10.1021/acs.jafc.0c02918.
59. Ravasio, D.; Wendland, J.; Walther, A. Major contribution of the Ehrlich pathway for 2-phenylethanol/rose flavor production in *Ashbya gossypii*. *FEMS Yeast Res* **2014**, *14*, 833-844, doi:10.1111/1567-1364.12172.
60. Derrick, S.; Large, P.J. Activities of the enzymes of the Ehrlich pathway and formation of branched-chain alcohols in Saccharomyces cerevisiae and Candida utilis grown in continuous culture on valine or ammonium as sole nitrogen source. *Microbiology* **1993**, *139*, 2783-2792, doi:10.1099/00221287-139-11-2783.
61. Kim, B.; Cho, B.R.; Hahn, J.S. Metabolic engineering of *Saccharomyces cerevisiae* for the production of 2-phenylethanol via Ehrlich pathway. *Biotechnol Bioeng* **2014**, *111*, 115-124, doi:10.1002/bit.24993.

62. Reygaert, W.C. An overview of the antimicrobial resistance mechanisms of bacteria. *AIMS Microbiol* **2018**, *4*, 482-501, doi:10.3934/microbiol.2018.3.482.
63. Baranova, N.; Nikaido, H. The baeSR two-component regulatory system activates transcription of the yegMNOB (mdtABCD) transporter gene cluster in Escherichia coli and increases its resistance to novobiocin and deoxycholate. *J Bacteriol* **2002**, *184*, 4168-4176, doi:10.1128/jb.184.15.4168-4176.2002.
64. Nagakubo, S.; Nishino, K.; Hirata, T.; Yamaguchi, A. The putative response regulator BaeR stimulates multidrug resistance of Escherichia coli via a novel multidrug exporter system, MdtABC. *J Bacteriol* **2002**, *184*, 4161-4167, doi:10.1128/jb.184.15.4161-4167.2002.
65. Hobbs, E.C.; Yin, X.; Paul, B.J.; Astarita, J.L.; Storz, G. Conserved small protein associates with the multidrug efflux pump AcrB and differentially affects antibiotic resistance. *Proc Natl Acad Sci U S A* **2012**, *109*, 16696-16701, doi:10.1073/pnas.1210093109.
66. Guan, N.; Liu, L. Microbial response to acid stress: mechanisms and applications. *Appl Microbiol Biotechnol* **2020**, *104*, 51-65, doi:10.1007/s00253-019-10226-1.
67. Olkhova, E.; Hunte, C.; Screpanti, E.; Padan, E.; Michel, H. Multiconformation continuum electrostatics analysis of the NhaA Na+/H+ antiporter of *Escherichia coli* with functional implications. *Proc Natl Acad Sci U S A* **2006**, *103*, 2629-2634, doi:10.1073/pnas.0510914103.
68. Fujisawa, M.; Ito, M.; Krulwich, T.A. Three two-component transporters with channel-like properties have monovalent cation/proton antiport activity. *Proc Natl Acad Sci U S A* **2007**, *104*, 13289-13294, doi:10.1073/pnas.0703709104.
69. Ferguson, G.P.; Nikolaev, Y.; McLaggan, D.; Maclean, M.; Booth, I.R. Survival during exposure to the electrophilic reagent *N*-ethylmaleimide in *Escherichia coli*: role of KefB and KefC potassium channels. *J Bacteriol* **1997**, *179*, 1007-1012, doi:10.1128/jb.179.4.1007-1012.1997.
70. Sohlenkamp, C.; Geiger, O. Bacterial membrane lipids: diversity in structures and pathways. *FEMS Microbiol Rev* **2016**, *40*, 133-159, doi:10.1093/femsre/fuv008.
71. Gowrishankar, J. Nucleotide sequence of the osmoregulatory proU operon of *Escherichia coli*. *J Bacteriol* **1989**, *171*, 1923-1931, doi:10.1128/jb.171.4.1923-1931.1989.
72. Haardt, M.; Kempf, B.; Faatz, E.; Bremer, E. The osmoprotectant proline betaine is a major substrate for the binding-protein-dependent transport system ProU of *Escherichia coli* K-12. *Mol Gen Genet* **1995**, *246*, 783-786, doi:10.1007/bf00290728.
73. Farr, S.B.; Kogoma, T. Oxidative stress responses in Escherichia coli and Salmonella typhimurium. *Microbiol Rev* **1991**, *55*, 561-585, doi:10.1128/mr.55.4.561-585.1991.
74. Takeda, Y.; Avila, H. Structure and gene expression of the *E. coli* Mn-superoxide dismutase gene. *Nucleic Acids Res* **1986**, *14*, 4577-4589, doi:10.1093/nar/14.11.4577.
75. Saporito, S.M.; Cunningham, R.P. Nucleotide sequence of the nfo gene of *Escherichia coli* K-12. *J Bacteriol* **1988**, *170*, 5141-5145, doi:10.1128/jb.170.11.5141-5145.1988.
76. Laviad-Shitrit, S.; Izhaki, I.; Whitman, W.B.; Shapiro, N.; Woyke, T.; Kyrpides, N.C.; Halpern, M. Draft genome of *Rosenbergiella nectarea* strain 8N4(T) provides insights into the potential role of this species in its plant host. *PeerJ* **2020**, *8*, e8822, doi:10.7717/peerj.8822.
77. Bagg, A.; Neilands, J.B. Ferric uptake regulation protein acts as a repressor, employing iron (II) as a cofactor to bind the operator of an iron transport operon in *Escherichia coli*. *Biochemistry* **1987**, *26*, 5471-5477, doi:10.1021/bi00391a039.
78. Seaver, L.C.; Imlay, J.A. Alkyl hydroperoxide reductase is the primary scavenger of endogenous hydrogen peroxide in *Escherichia coli*. *J Bacteriol* **2001**, *183*, 7173-7181, doi:10.1128/jb.183.24.7173-7181.2001.
79. Almirón, M.; Link, A.J.; Furlong, D.; Kolter, R. A novel DNA-binding protein with regulatory and protective roles in starved *Escherichia coli*. *Genes Dev* **1992**, *6*, 2646-2654, doi:10.1101/gad.6.12b.2646.

80. Nair, S.; Finkel, S.E. Dps protects cells against multiple stresses during stationary phase. *J Bacteriol* **2004**, *186*, 4192-4198, doi:10.1128/jb.186.13.4192-4198.2004.
